# A *DERL3*-associated defect in the degradation of SLC2A1 mediates the Warburg effect

**DOI:** 10.1038/ncomms4608

**Published:** 2014-04-03

**Authors:** Paula Lopez-Serra, Miguel Marcilla, Alberto Villanueva, Antonio Ramos-Fernandez, Anna Palau, Lucía Leal, Jessica E. Wahi, Fernando Setien-Baranda, Karolina Szczesna, Catia Moutinho, Anna Martinez-Cardus, Holger Heyn, Juan Sandoval, Sara Puertas, August Vidal, Xavier Sanjuan, Eva Martinez-Balibrea, Francesc Viñals, Jose C. Perales, Jesper B. Bramsem, Torben F. Ørntoft, Claus L. Andersen, Josep Tabernero, Ultan McDermott, Matthew B. Boxer, Matthew G. Vander Heiden, Juan Pablo Albar, Manel Esteller

**Affiliations:** 1 Cancer Epigenetics and Biology Program (PEBC), Bellvitge Biomedical Research Institute (IDIBELL), L'Hospitalet, Barcelona, 08908 Catalonia, Spain; 2 Proteomics Unit, Spanish National Biotechnology Centre (CNB), CSIC, 28049 Madrid, Spain; 3 Translational Research Laboratory, Catalan Institute of Oncology (ICO), Bellvitge Biomedical Research Institute (IDIBELL), L'Hospitalet, Barcelona, 08908 Catalonia, Spain; 4 Proteobotics SL, Spanish National Biotechnology Centre (CNB), Darwin 3, 28049 Madrid, Spain; 5 Bellvitge Biomedical Research Institute (IDIBELL), Department of Pathology, Bellvitge University Hospital, L'Hospitalet, Barcelona, 08908 Catalonia, Spain; 6 Medical Oncology Service, Catalan Institute of Oncology (ICO), l'Institut d'Investigació en Ciències de la Salut Germans Trias i Pujol (IGTP), Hospital Germans Trias I Pujol, Badalona, Barcelona, 08916 Catalonia, Spain; 7 Department of Physiological Sciences II, School of Medicine, University of Barcelona, 08036 Barcelona, Spain; 8 Department of Molecular Medicine, Aarhus University Hospital, Brendstrupgaardsvej 100, Aarhus N, DK-8200 Aarhus, Denmark; 9 Medical Oncology Department, Vall d’Hebron University Hospital, Barcelona, 08035 Catalonia, Spain; 10 Cancer Genome Project, Wellcome Trust Sanger Institute, Hinxton CB10 1SA, UK; 11 National Center for Advancing Translational Sciences, National Institutes of Health, Rockville, Maryland 20891-4874, USA; 12 Koch Institute for Integrative Cancer Research, Massachusetts Institute of Technology, Cambridge, Massachusetts 02139, USA; 13 Department of Medical Oncology, Dana-Farber Cancer Institute, Boston, Massachusetts 02215, USA; 14 Institucio Catalana de Recerca i Estudis Avançats (ICREA), Barcelona, 08010 Catalonia, Spain

## Abstract

Cancer cells possess aberrant proteomes that can arise by the disruption of genes involved in physiological protein degradation. Here we demonstrate the presence of promoter CpG island hypermethylation-linked inactivation of *DERL3* (Derlin-3), a key gene in the endoplasmic reticulum-associated protein degradation pathway, in human tumours. The restoration of *in vitro* and *in vivo* DERL3 activity highlights the tumour suppressor features of the gene. Using the stable isotopic labelling of amino acids in cell culture workflow for differential proteome analysis, we identify SLC2A1 (glucose transporter 1, GLUT1) as a downstream target of DERL3. Most importantly, SLC2A1 overexpression mediated by *DERL3* epigenetic loss contributes to the Warburg effect in the studied cells and pinpoints a subset of human tumours with greater vulnerability to drugs targeting glycolysis.

## Abstract (web summary)

Defective proteins or functional proteins that are no longer needed can be degraded in the endoplasmic reticulum. In this study, Lopez-Serra *et al.* show that DERL3, which is involved in protein degradation in the endoplasmic reticulum, is aberrantly silenced in cancer, leading to activation of a glucose transporter and dysregulated glycolysis.

## Graphical abstract

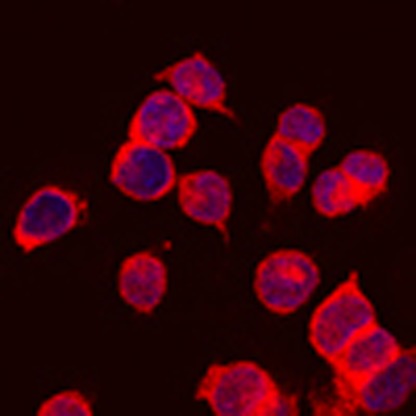

Physiological cellular activity requires a precise control of the proteome^1^. However, cancer cells have a distorted protein profile^2^. It is likely that intrinsic defects in protein homoeostasis participate in human tumorigenesis, such as alterations in *de novo* protein synthesis^3^, or defects in protein degradation, such as mutations in the von Hippel–Lindau ubiquitin ligase complex^4^. In this last case, the ubiquitin proteasome system (UPS) targets a variety of proteins, including functional proteins that are no longer needed^5^. Without appropriate protein homoeostasis maintained by the UPS, healthy cells can undergo malignant transformation^6^, and this observation has been therapeutically exploited by the development of proteasome inhibitors as anticancer agents^6^. Many of the cytoplasm UPS-degraded proteins are retrotranslocated from the endoplasmic reticulum (ER)^7^. In this regard, ER possesses a quality control mechanism, termed the ER-associated degradation mechanism (ERAD), that is induced in response to ER stress by a transcriptional program, known as the unfolded protein response, which leads to the accelerated degradation of unfolded proteins^8^,^9^. Within the ERAD pathway, the Derlin proteins^10^,^11^,^12^,^13^ play a critical role. It has been proposed that Derlins form an export channel in the membrane of the ER through which the ERAD substrates pass to reach the proteasome that is to be degraded^10^,^11^. Of the three members of the Derlin family (*DERL1*, *DERL2* and *DERL3*), *DERL1* is the most widely studied^13^, but all three are active ERAD proteins under conditions of protein stress^12^.

Although genetic alterations in Derlins have not been described in tumoral cells, transcriptional inactivation by CpG island promoter hypermethylation is an alternative mechanism for inactivating genes in human cancer^14^,^15^. Herein, we have characterized the presence of *DERL3* epigenetic inactivation in tumorigenesis and identified solute carrier 2A (SLC2A1) (glucose transporter 1, GLUT1) as a DERL3-mediated target for degradation. *SLC2A1* contributes to the tumoral cell metabolism reprogramming to generate energy by glycolysis even under normal oxygen concentrations (the Warburg effect) and to a different sensitivity to glycolysis inhibitors. The observed SLC2A1 overexpression induced by *DERL3* epigenetic loss generates a Warburg effect in the studied cells and it highlights a group of human tumours that are more sensitive to drugs targeting glycolysis.

## Results

### *DERL3* CpG island hypermethylation leads to gene inactivation

**Figure 1:**
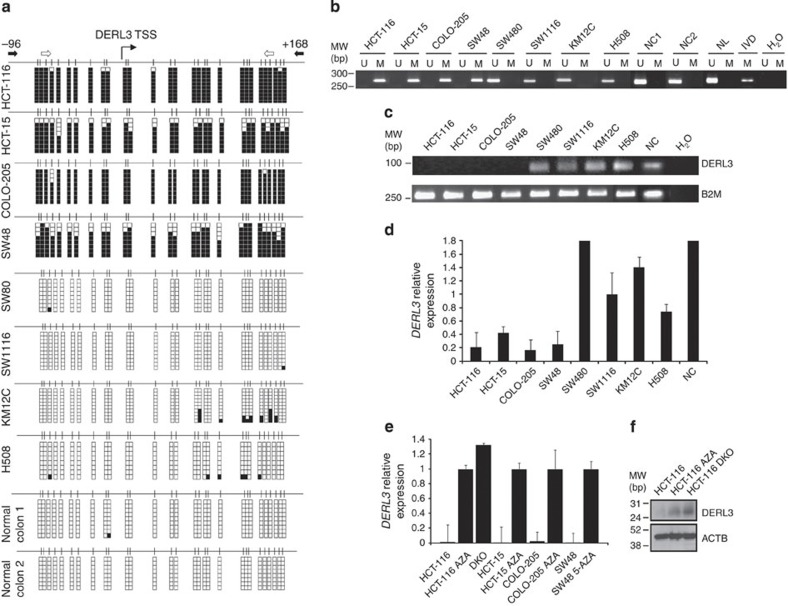
DNA methylation-associated transcriptional silencing of *DERL3*. (**a**) Bisulphite genomic sequencing of *DERL3* promoter CpG Island. CpG dinucleotides are represented as short vertical lines and the transcriptional start site (TSS) is represented as a long black arrow. The locations of the bisulphite genomic sequencing primers are indicated by black arrows. At least eight single clones are shown for each sample. Presence of a methylated or unmethylated cytosine is indicated by a black or a white square, respectively. (**b**) Methylation-specific PCR (MSP) analyses. The locations of the MSP primers are indicated by white arrows. The presence of a band under the U or M lanes indicates unmethylated or methylated sequences, respectively. Normal lymphocyte/colon (NC and NL) and *in vitro* methylated DNA (IVD) are shown as positive controls for the unmethylated and methylated sequences, respectively. Expression levels of the *DERL3* transcript were determined by semiquantitative PCR (**c**) and real-time reverse transcription-PCR (**d**). (**e**) The expression of *DERL3* RNA transcript was restored in the HCT-116, HCT-15, COLO-205 and SW48 cells by treatment with the demethylating drug 5-aza-2-deoxycytidine (AZA). Genetic disruption of the two major DNA methyltransferases *DNMT1* and *DNMT3B* (DKO cells) also restored *DERL3* expression in HCT-116 cells. Data shown represent mean±s.e.m. of biological triplicates. (**f**) Protein levels of DERL3 were recovered after AZA treatment in HCT-116 cells and in DKO cells.

*DERL1*, *DERL2* and *DERL3* are gene candidates for hypermethylation-associated inactivation in human cancer because a 5′-CpG island is located around their transcription start sites (Fig. 1a and Supplementary Figs 1 and 2). To analyse the DNA methylation status of the promoter-associated CpG islands, we screened 28 human colorectal cancer cell lines using bisulphite genomic sequencing analyses of multiple clones (Fig. 1a and Supplementary Fig. 1) and by using a DNA methylation microarray approach^16^ (Supplementary Fig. 2). *DERL1* and *DERL2* 5′-end CpG island were unmethylated in all cases, but *DERL3* CpG island promoter hypermethylation was found in four colorectal cancer cell lines: HCT-116, HCT-15, COLO-205 and SW48 (Fig. 1a and Supplementary Fig. 2). All normal colorectal mucosa tissues analysed were unmethylated at the DERL3 promoter (Fig. 1a and Supplementary Fig. 2). There were no significant differences between the DERL3-methylated and -unmethylated groups of colorectal cancer cell lines for their expression pattern of DNA methyltransferases (Supplementary Fig. 3) or their basal cellular growth rates (Supplementary Fig. 3). The CpG island methylation patterns described for *DERL3* were confirmed by methylation-specific PCR (Fig. 1b).

Having noted the *DERL3* promoter hypermethylation in cancer cell lines, we assessed its association with the putative transcriptional inactivation of the *DERL3* gene at the RNA and protein levels. The cancer cell lines HCT-116, HCT-15, COLO-205 and SW48, hypermethylated at the *DERL3* CpG island, had minimal expression of the *DERL3* RNA transcript, as determined by conventional and quantitative reverse transcription-PCR (Fig. 1c,d). By contrast, four colorectal cancer cell lines unmethylated at the *DERL3* promoter (SW480, SW1116, KM12C and H508) expressed *DERL3* RNA (Fig. 1c,d). We established a further link between *DERL3* CpG island hypermethylation and its gene silencing by treating the methylated cell lines with a DNA-demethylating agent. treatment of the HCT-116, HCT-15, COLO-205 and SW48 cell lines with 5-aza-2-deoxycytidine restored DERL3 expression (Fig. 1e). These results were confirmed in the isogenic HCT-116 cell line in which the two major DNA methyltransferases, *DNMT1* and *DNMT3B*, had been genetically disrupted (DKO)^17^. We observed that the *DERL3* CpG island was unmethylated in double knock-out (DKO) cells and, most importantly, that *DERL3* transcription was restored (Fig. 1e). Western blot analysis confirmed the absence of DERL3 protein expression in HCT-116 cells and its recovery on treatment with the demethylating drug and DKO cells (Fig. 1f).

### DERL3 has tumour suppressor-like properties in cancer cells

Having observed the CpG island hypermethylation-associated silencing of *DERL3* in colorectal cancer cells, we then assayed the ability of *DERL3* to function as a suppressor of tumour growth, using *in vitro* and *in vivo* approaches.

**Figure 2:**
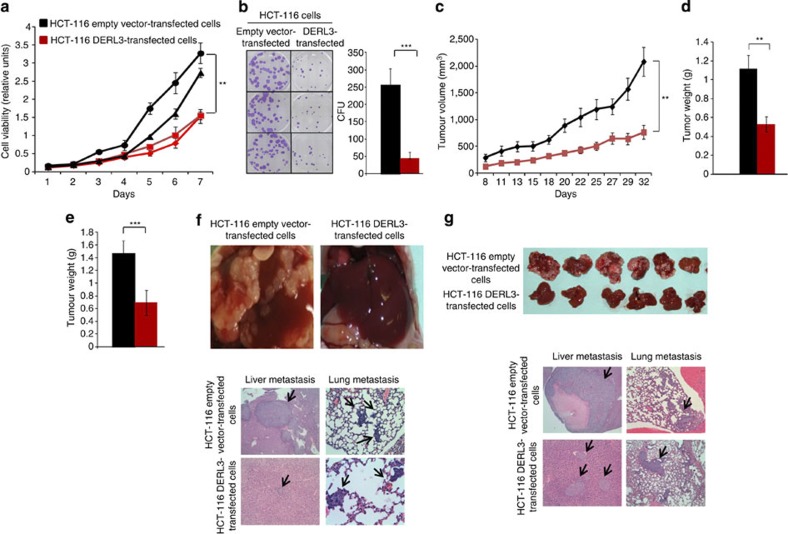
Restoration of *DERL3* expression has tumour suppressor-like properties *in vitro* and *in vivo*. (**a**) Cell proliferation differences between DERL3-expressing clones and the corresponding controls determined by the MTT assay and monitored for 7 days; *P*-values for exact binomial test (**P<0.01). (**b**) Colony formation assay. The number of colonies formed 2 weeks after seeding 1,000 cells on 35 mm plates and maintained in selection media were quantified and plotted; *P*-values for Student's *t*-tests (***P<0.005). HCT-116 cells transfected with DERL3 or empty vector were injected into the flanks of severe combined immunodeficiency (SCID) mice. Tumour volume was measured for 30 days (**c**) and tumour weight determined at the end of that period (**d**). *P*-values are those associated with Mann–Whitney *U*-tests (**P<0.01). (**e**) Orthotopic growth study implanting equal tumour pieces from the subcutaneous model in the colon tract. Tumour weight was measured at 30 days. *P*-values are those associated with Mann–Whitney *U*-tests (***P<0.005). (**f**) Illustrative examples of differential peritoneal colonization and hematoxylin and eosin stain (H&E) staining of colonized liver and lungs are shown. (**g**) Illustrative surgery samples and H&E staining of colonized liver and lung after spleen injection are shown.

For the *in vitro* approach, we stably transfected the HCT-116 cell line, hypermethylated and silenced for *DERL3*, with the *DERL3* gene (DERL3-FLAG pIRES2-eGFP expression vector). The efficiency of transfection was assessed by measuring *DERL3* expression by quantitative reverse transcription-PCR, western blot and immunocytofluorescence (Supplementary Fig. 4). On transfection of *DERL3* in the hypermethylated HCT-116 colorectal cancer cell line, the cells proved to be significantly less proliferative in the 3-(4,5-dimethylthiazol-2-yl)-2,5-diphenyltetrazolium bromide (MTT) assay (Fig. 2a), and had a significantly lower percentage colony formation density (Fig. 2b) than empty vector (pIRES2-eGFP)-transfected cells.

For the *in vivo* approach, we used tumour and metastasis formation assays in nude mice. First, HCT-116 cells transfected with either the empty or the *DERL3* vector were subcutaneously injected into nude mice. Tumours originated from DERL3-transfected HCT-116 cells had a significantly lower volume and weight than empty vector-transfected-derived tumours (Fig. 2c,d). We confirmed these data in HCT-15 cells, another *DERL3*-hypermethylated colon cancer cell line, where *DERL3* transfection also induced significantly smaller and lighter tumours (Supplementary Fig. 5). Second, we performed an orthotopic growth study, implanting equal-sized tumour pieces from the subcutaneous model in the colon tract. We observed that orthotopic DERL3-transfected tumours were significantly smaller than the empty vector-transfected tumours (Fig. 2e). Interestingly, although all (12 out of 12, 100%) empty vector orthotopics were able to locally colonize to the peritoneal cavity of the mice, only one (8%) of the DERL3-transfected tumours had this ability (Fig. 2f; Table 1). With respect to blood dissemination, only 17% of the orthotopic DERL3-transfected tumours yielded metastasis in the liver and the lung compared with the existence of liver and lung metastasis in 83% of the orthotopic empty vectors (Table 1). Finally, the distant inhibitory dissemination activity of DERL3 was measured in athymic mice by direct spleen injection and analysis of metastasis formation. Whereas numerous metastatic nodules developed in the liver and lung following injection of empty vector-transfected HCT-116 cells, a reduction in metastasis formation was observed with the same number of DERL3-transfected HCT-116 cells over the same period (Fig. 2g; Table 1). The same phenomenon was observed for DERL3-transfected HCT-15 cells (Supplementary Fig. 5). Overall, the findings presented here suggest tumour suppressor and dissemination inhibitor roles for DERL3.

### SLC2A1 as a target of DERL3-mediated degradation

**Figure 3:**
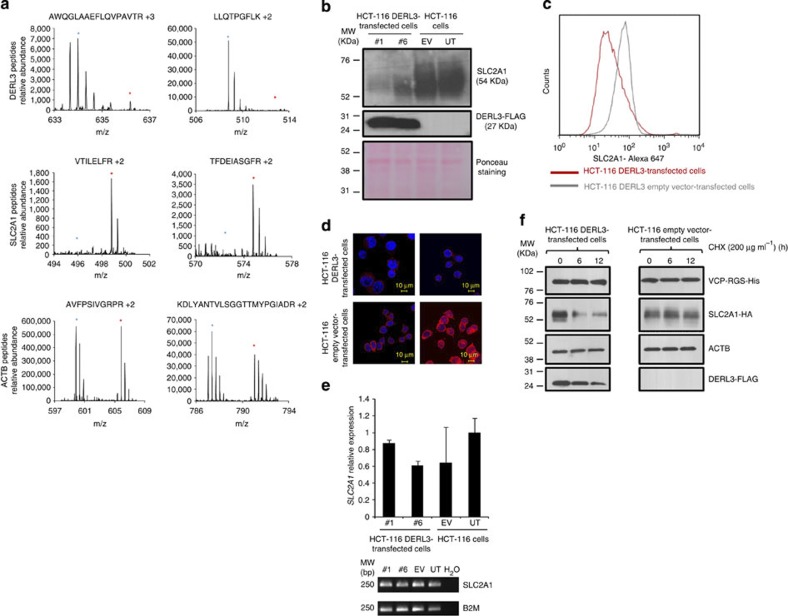
Stable isotopic labelling of amino acids in cell culture identifies SLC2A1 as a candidate target for DERL3-mediated protein degradation. (**a**) Mass spectrometry spectra obtained by the SILAC approach in empty vector (red asterisk)- and DERL3 (blue asterisk)-transfected cells: upper column, recovery of DERL3 protein expression in HCT-116-transfected cells confirmed by multiple SILAC light signals originated by two tryptic peptides; middle column, strong downregulation of signals from two SLC2A1 peptides (VTILELFR and TFDEIASGFR with log2 fold changes of 4.22 and 3.56, respectively) in DERL3-transfected cells; lower column, signals from peptides of a non-DERL3 differentially regulated protein (ACTB) show light:heavy signal patterns close to the expected 1:1 proportion. (**b**) Western blot, (**c**) cell cytometry and (**d**) immunofluorescence show downregulation of the SLC2A1 protein in DERL3 stably transfected HCT-116 cells in comparison with empty vector-transfected cells. (**e**) Semiquantitative and quantitative reverse transcription-PCR shows that DERL3-dependent downregulation of SLC2A1 occurred at the protein level and was not associated with a difference in *SLC2A1* mRNA levels. Data shown represent mean±s.e.m. of biological triplicates. (**f**) Treatment of DERL3-FLAG and pIRES2-eGFP HCT-116-transfected cells with cycloheximide in a time-course experiment followed by western blotting shows that SLC2A1 protein degradation occurs only in the presence of DERL3.

The proteins of the Derlin family form export channels in the membrane of the ER through which the ERAD substrates pass to reach the proteasome^10^,^11^. This prompted us to consider whether the induction of degradation in growth-promoting proteins was the mechanism underlying tumour and metastasis suppression on *DERL3* transfection. No specific cellular targets for DERL3-associated degradation have been identified to date in this respect. To identify DERL3 downstream protein effectors with a potential role in cellular transformation, we performed stable isotopic labelling of amino acids in cell culture (SILAC)^18^ in empty vector-transfected HCT-116 cells (with *DERL3* hypermethylation-associated loss) in comparison with the *DERL3* stably transfected HCT-116 cells, in which we had restored *DERL3* RNA and protein expression, as described above. HCT-116 empty vector (pIRES2-eGFP) and *DERL3* (DERL3-FLAG pIRES2-eGFP)-transfected cells were grown in heavy and light culture media, respectively, and we fractionated the cell extracts to enrich the samples in proteins from the secretory pathway (ER, Golgi apparatus and cellular membrane) and organelles. The SILAC analyses identified 33 proteins represented by at least two peptides that were significantly downregulated on *DERL3* transfection in HCT-116 cells (Supplementary Table 1). Supplementary Table 1 lists several members of the CCT complex, but at least for CCT5, the downregulation of this protein was an example of an indirect effect of DERL3 re-expression because its mRNA was also downregulated (Supplementary Fig. 6). Twenty-seven proteins were found upregulated, probably due to indirect or compensatory mechanisms (Supplementary Table 2). The distribution of all quantified proteins by SILAC is shown in a histogram in Supplementary Fig. 6. Most importantly, we observed that the first protein significantly depleted in *DERL3* stably transfected cells corresponded to SLC2A1 (glucose transporter 1, GLUT1) (Fig. 3a). Most cancer cells undergo a shift in metabolism away from oxidative phosphorylation towards aerobic glycolysis, a phenomenon termed the Warburg effect^19^,^20^,^21^. Aerobic glycolysis depends on increased glucose uptake via SLC2A1, and there is potential use of small molecules to target glucose-dependent metabolic vulnerabilities of transformed cells^22^,^23^. Thus, we further characterized SLC2A1 as a putative target of DERL3-mediated degradation.

First, we confirmed by western blot, cell cytometry and immunofluorescence analysis (Fig. 3b–d) that the SLC2A1 protein was downregulated in *DERL3* stably transfected HCT-116 cells relative to empty vector-transfected cells. We also proved that DERL3-dependent downregulation occurred at the protein level and was not associated with a difference in *SLC2A1* transcriptional regulation by using conventional and quantitative reverse transcription-PCR to show that *SLC2A1* RNA levels were equivalent in *DERL3* and empty vector HCT-116-transfected cells (Fig. 3e). We confirmed these data in HCT-15 cells where DERL3 transfection also induced downregulation of the SLC2A1 protein, but not of the *SLC2A1* mRNA (Supplementary Fig. 5). To confirm the specific protein degradation of SLC2A1 by DERL3, we transfected both HCT-116 cell lines (DERL3-FLAG pIRES2-eGFP and pIRES2-eGFP cells) with the SLC2A1-HA recombinant protein (Fig. 3f). These cells were also co-transfected with the VCP ATPase protein (VCP-RGS-His recombinant protein), which is involved in the ATP supply for the Derlin-associated export channels of the ER membrane^13^,^24^, to avoid any energetic limitation of the assay. Treating these cells with cycloheximide in a time-course experiment followed by western blotting of the cell lysates showed that SLC2A1 protein degradation occurred only in the presence of DERL3 (Fig. 3f). We ruled out the possibility that *DERL3* transfection induced SLC2A1 downregulation within the context of an unspecific global protein degradation response: no differences in ubiquitin levels were observed between HCT-116-, DERL3-FLAG- and pIRES2-eGFP-transfected cells (Supplementary Fig. 7). We also observed by immunoprecipitation the interaction between DERL3 and SLC2A1 and the colocalization of both proteins in the ER (Supplementary Fig. 7). Finally, we found that SLC2A1 exerted an important role in the tumour suppressor effects mediated by DERL3. The knockdown of *SLC2A1* expression in HCT-116 cells reduced cellular proliferation, and we observed that *SLC2A1* transfection in our DERL3-transfected HCT-116 cells caused an increase in cellular proliferation (Supplementary Fig. 8). Overall, these data indicate that the DNA methylation-associated silencing of *DERL3* in HCT-116 colorectal cancer cells causes a pro-tumorigenic upregulation of the SLC2A1 protein that can be reversed by restoring DERL3 activity.

### SLC2A1-impaired degradation sensitizes to glycolysis drugs

**Figure 4:**
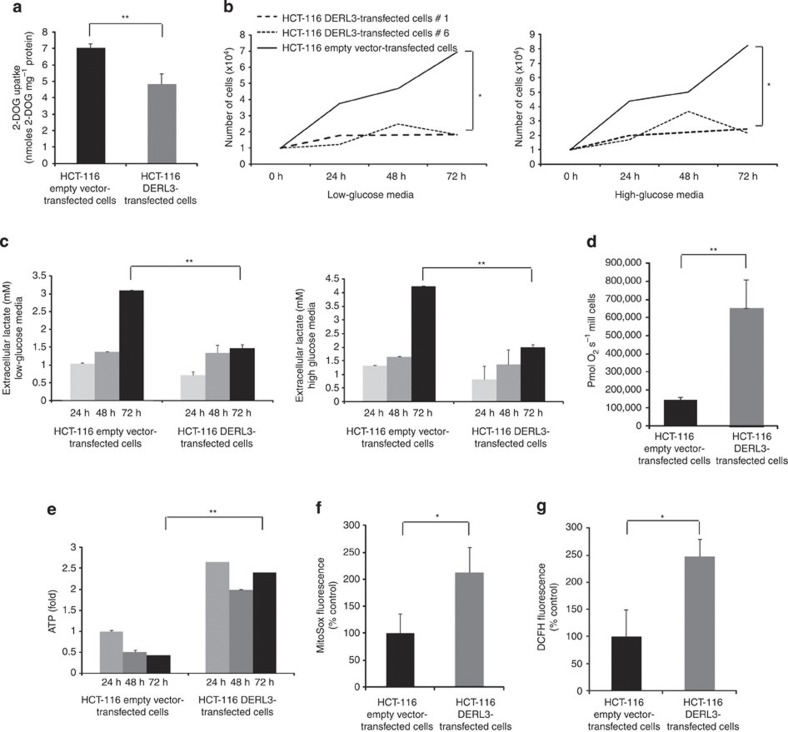
SLC2A1 upregulation mediated by *DERL3* loss causes glucose metabolism shifts. (**a**) 2-deoxyglucose uptake is diminished on transfection of *DERL3* in HCT-116 cells. Data shown represent the mean±s.d. *P*-value obtained by Student’s *t*-test (**P<0.01). (**b**) Glucose-dependent growth is observed in empty vector-transfected HCT-116 cells, but not in DERL3-transfected cells in low- and high-glucose content media. *P*-values are those associated with Student’s *t*-test (*P<0.05). (**c**) Lactate production is depleted on transfection of *DERL3* in HCT-116 cells in low- and high-glucose content media. Data shown represent the mean±s.d. *P*-values are those associated with Student’s *t*-test (**P<0.01). (**d**) Oxygen consumption rate is higher in HCT-116 DERL3-transfected cells (**P<0.01) and is also associated with a greater production of ATP (**P<0.01) (**e**) and ROS at the mitochondrial (**f**) and total (**g**) levels (*P<0.05 in both cases). Data shown represent mean±s.e.m. of biological duplicates. All *P*-values were according to Student’s *t*-test.

*SLC2A1* is a ubiquitously expressed member of the SLC2A family of glucose transporters, rate-limiting step proteins in glucose metabolism^25^,^26^. In this regard, we found that DERL3-transfected HCT-116 cells experimented a diminished cellular glucose uptake in comparison with empty vector-transfected cells (Fig. 4a). The same phenomenon was observed on recovery of DERL3 expression in HCT-15 cells (Supplementary Fig. 5). Importantly, a characteristic feature of cancer cells is the increased dependence on glucose to fuel aerobic glycolysis, which produces a large amount of lactate even in a well-oxygenated environment^19^,^20^,^21^. Thus, we wondered whether SLC2A1 expression levels mediated by DERL3 degradation affected cancer cell glucose-dependent growth and lactate production. To address this, we cultured HCT-116 empty vector and DERL3-transfected cells in pyruvate and glutamine-free media and supplemented the media with low or high glucose content. We observed that a significant increase in the proliferation of HCT-116 empty vector-transfected cells occurred over time in low- and high-glucose content media (Fig. 4b). However, in *DERL3* stably transfected cells, where SLC2A1-enhanced degradation occurs, glucose supplementation was unable to increase basal proliferation over time (Fig. 4b). The distinct dependence on glucose for cell proliferation was also reflected in the rate of lactate production. We observed significantly lower extracellular lactate production in DERL3-transfected cells, under low- or high-glucose conditions, compared with the high level of production in HCT-116 empty vector-transfected cells (Fig. 4c). To avoid differences in lactate production due to the cell growth rate, lactate concentration was normalized using the total protein content of the cultures. In addition, *DERL3* stably transfected cells had a higher basal oxygen consumption rate than empty vector-transfected HCT-116 cells (Fig. 4d). The pronounced difference in oxygen consumption was also associated with increased cellular ATP levels in the DERL3-transfected HCT-116 cells, supporting a shift from aerobic glycolysis to oxidative phosphorylation on DERL3 restoration (Fig. 4e). This was also associated with a higher level of production of mitochondrial reactive oxygen species (ROS) (Fig. 4f) and intracellular total ROS (Fig. 4g). Interestingly, increases in the cellular concentration of ROS inhibit the glycolytic enzyme pyruvate kinase-M2 (PKM2) through oxidation of Cys^358^ (ref. 27), adding an additional regulatory layer of glycolysis mediated by DERL3.

**Figure 5:**
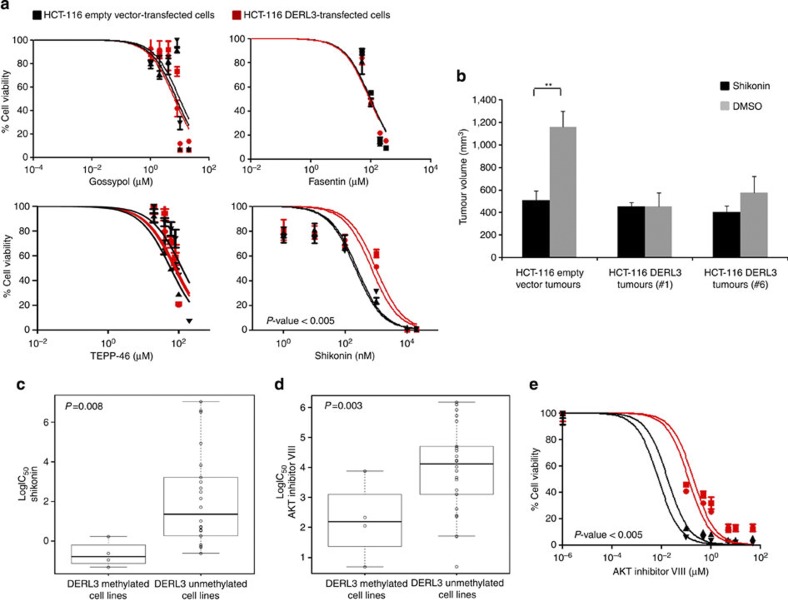
DERL3 loss and sensitivity to glycolysis inhibitors. (**a**) MTT assays for cell viability of DERL3 and empty vector-transfected HCT-116 on the use of four drugs that target cancer metabolism. Empty vector HCT-116 cells (*DERL3*-deficient) were significantly more sensitive to the PKM2 inhibitor shikonin. Data shown represent mean±s.d. (**b**) Shikonin significantly reduces tumour growth in the empty vector HCT-116-derived tumours generated in nude mice, but does not affect the growth rate of tumours derived from HCT-116 DERL3-transfected cells. Ten mice were used for each condition. Data shown represent mean±s.d. (**c**) *DERL3* promoter CpG island hypermethylation is significantly associated with enhanced sensitivity to shikonin in the Sanger panel of colorectal cancer cell lines. (**d**) The presence of *DERL3* promoter CpG island hypermethylation is significantly associated with enhanced sensitivity to the AKT inhibitor VIII in the Sanger set of colorectal cancer cell lines. The box plots display the distribution of IC_50_ values with the central solid line representing the median and the limits of the box, the upper and lower quartiles. The whiskers represent the minimum and maximum values excluding outliers (<1.5 × the interquartile range). (**e**) Empty vector HCT-116 cells were significantly more sensitive to the antiproliferative effect of the AKT inhibitor VIII than were DERL3-transfected cells.

The dependence of *DERL3* epigenetically silenced cells on glucose prompted us to examine whether these cells were more sensitive to small drugs that target cancer metabolism, particularly glycolysis. Thus, we analysed the antiproliferative effects on HCT-116 empty vector- and DERL3-transfected cells of four drugs: a lactate dehydrogenase inhibitor (gossypol)^28^, a general inhibitor of glucose transport N-[4-chloro-3-(trifluoromethyl)phenyl]-3-oxobutanamide (fasentin)^29^ and an activator (TEPP-46)^30^ and an inhibitor (shikonin)^31^,^32^ of the aforementioned enzyme PKM2. No differences were observed, except for shikonin (Fig. 5a). It was very interesting the discovery associated with the PKM2 inhibitor shikonin that empty vector HCT-116 cells (*DERL3*-deficient) were significantly more sensitive to the antiproliferative effects of the drug than were DERL3-transfected cells (Fig. 5a). The finding that *DERL3*-hypermethylated cancer cells were highly sensitive to shikonin, and that restoration of *DERL3* expression induced resistance to this drug was further confirmed by an MTT growth inhibition curve, the SRB (Sulforhodamine B) assay and colony formation experiments (Supplementary Fig. 9). We translated these results to an *in vivo* animal-tumour model. We generated subcutaneous tumours in nude mice using the empty vector- or DERL3-transfected HCT-116 cells and treated the mice with the PKM2 inhibitor shikonin. Shikonin treatment significantly reduced the volume of the empty vector-transfected HCT-116 cells, but the DERL3-transfected tumours did not show any significant difference between the shikonin and carrier solution treatments (Student’s *t*-test, *P*=0.003) (Fig. 5b).

The widely recognized role of *PKM2* in human tumorigenesis^33^ prompted us to determine how generally *DERL3* epigenetic inactivation confers sensitivity on the PKM2 inhibitor. To examine this, we compared the *DERL3* promoter CpG island methylation status in our 28 colorectal cancer cell lines (Supplementary Fig. 2) with the corresponding sensitivity to shikonin that had already been determined for 22 of them^34^. In this context, we observed that *DERL3* promoter CpG island hypermethylation was also significantly associated with enhanced sensitivity to shikonin (multivariate analysis of variance test, false discovery rate (FDR)<0.01, *P*=0.008) (Fig. 5c). No significant equivalent sensitivity was noted for other cancer metabolic drugs such as PIK3CA (GDC0941 and AZD6482), GSK3A/B (SB 216763) inhibitors and PRKAA2 (AICAR and metformin) agonists (Supplementary Fig. 10). However, one remarkable exception was the AKT inhibitor VIII, for which the presence of *DERL3* promoter CpG island hypermethylation was significantly associated with enhanced sensitivity in the panel of 22 colorectal cancer cell lines (multivariate analysis of variance test, FDR<0.01, *P*=0.003) (Fig. 5d). Following this observation, we treated our HCT-116 experimental model with this agent and confirmed the finding in this setting: the empty vector HCT-116 cells (DERL3-deficient) were significantly more sensitive to the antiproliferative effects of the AKT inhibitor VIII than were the DERL3-transfected cells (Fig. 5e). These results are consistent with the finding that the AKT signalling cascade activates the Warburg effect by inducing *PKM2* (ref. 35), for which reason the AKT inhibitor VIII can be considered an indirect PKM2 inhibitor.

Overall, the findings suggest that *DERL3*-hypermethylated cancer cells undergo glucose-dependent growth mediated by SLC2A1 that renders them highly sensitive to particular glycolysis inhibitors.

### *DERL3* epigenetic loss occurs in different human malignancies

**Figure 6:**
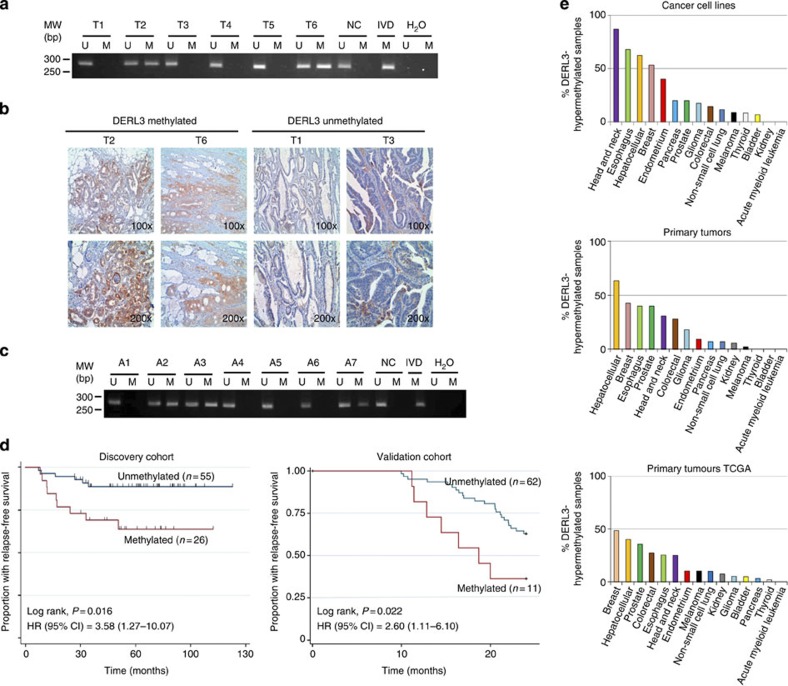
*DERL3* hypermethylation in human colorectal tumorigenesis and primary malignancies. (**a**) Methylation-specific PCR analyses for *DERL3* in primary human colorectal tumours. The presence of a band under the U or M lanes indicates unmethylated or methylated sequences, respectively. Normal colon (NC) and *in vitro* methylated DNA (IVD) are shown as positive controls for the unmethylated and methylated sequences, respectively. (**b**) Illustrative immunohistochemical examples demonstrate that SLC2A1 overexpression in human primary colorectal tumours is associated with *DERL3* promoter CpG island hypermethylation. (**c**) Methylation-specific PCR analyses for *DERL3* in benign colorectal adenomas. (**d**) The presence of *DERL3* hypermethylation in primary colorectal tumours is significantly associated with shorter relapse-free survival in two cohorts of patients (discovery and validation groups). (**e**) Percentage of hypermethylated *DERL3* samples in cancer cell lines, primary tumours from our study and data available from The Cancer Genome Atlas (TCGA) consortium.

The presence of *DERL3* cancer-specific promoter CpG island hypermethylation is not an *in vitro* phenomenon restricted to colorectal cancer cell lines. The analyses of a large collection of primary human colorectal adenocarcinomas showed that *DERL3* promoter hypermethylation was common. In a first set of colon tumours (*n*=91), we found *DERL3* hypermethylation determined by methylation-specific PCR in 24% (22 of 91) of patients (Fig. 6a). We confirmed the existence of *DERL3* CpG island methylation in a second set of primary colorectal adenocarcinomas (*n*=128) using the DNA methylation microarray platform^16^, in which it was found in 28% (36 of 128) of patients (Supplementary Table 3). Most importantly, as we also noted in the HCT-116 colon cancer cell line model, we observed that the presence of *DERL3* CpG island hypermethylation in primary colorectal tumours was associated with overexpression of the SLC2A1 protein determined by immunohistochemistry (Fisher’s exact test, *P*=0.05, *n*=16) (Fig. 6b). In this set of cases, both *DERL3*-hypermethylated cases (100%) had a high frequency of SLC2A1-overexpressing cells, while in the unmethylated cases enhanced SLC2A1 expression was found in only 14% (2 of 14) of patients.

We found that *DERL3* hypermethylation represents an early lesion. When we examined the *DERL3* CpG island methylation status in colorectal adenomas (*n*=12), a lesion that is a precursor of invasive colorectal tumours, we observed that *DERL3* hypermethylation was already present in 33% (4 of 12) of these samples (Fig. 6c). Interestingly, we also noted that *DERL3* hypermethylation was of prognostic value. Comparing the *DERL3* methylation data against the clinicopathological values of colorectal cancer patients, for 81 cases for which this information was available, we found that *DERL3* CpG island hypermethylation was associated with shorter relapse-free survival (Kaplan–Meier analysis, log rank, *P*=0.016; hazard ratio 3.58; 95% CI: 1.27–10.07) (Fig. 6d). We confirmed these data in a validation cohort of 73 colorectal cancer cases where the presence of *DERL3* hypermethylation assessed by methylation specific was also associated with shorter relapse-free survival (Kaplan–Meier analysis, log rank, *P*=0.022; hazard ratio 2.60; 95% CI: 1.11–6.10). All the studied tumours were microsatellite stable and only one, a *DERL3*-methylated case, had a *BRAF* mutation.

*DERL3* epigenetic silencing was not a unique feature of colorectal cancer. Using the aforementioned microarray platform in a large set of human cancer cell lines (*n*=346) from 14 tumour types, we observed that *DERL3* promoter CpG island methylation was commonly found in solid tumours, such as head and neck, breast, liver, oesophageal, prostate and lung carcinomas (Fig. 6e and Supplementary Table 3), but not in leukaemia. In these non-colorectal cancer cell line groups, we also confirmed that the presence of *DERL3* CpG island hypermethylation was associated with transcriptional silencing, while unmethylated cell lines expressed the *DERL3* transcript (Supplementary Fig. 11).

Most importantly, the CpG island hypermethylation of *DERL3* was not restricted to the cultured cells of these other tumour types, because the DNA methylation microarray approach applied to 1,186 human primary malignancies corresponding to the 14 tissue types revealed that *DERL3* CpG island hypermethylation was also commonly found in similar tumour types than those of the cancer cell lines (Fig. 6e and Supplementary Table 3). We also confirmed the absence of *DERL3* hypermethylation (Supplementary Table 4) in a set of corresponding normal tissues (*n*=102) from these locations, in addition to colon mucosa (*n*=17). Most importantly, using the same microarray platform, we found a similar distribution of *DERL3* hypermethylation among the primary tumour types when we analysed the public DNA methylation data from The Cancer Genome Atlas efforts ( https://tcga-data.nci.nih.gov/tcga/); (Fig. 6e and Supplementary Table 3). Thus, *DERL3* hypermethylation occurs in a broad spectrum of human malignancies that might respond to glycolysis inhibitors.

## Discussion

Cancer cells are extremely sensitive to glucose concentration changes, and glucose deprivation can induce growth inhibition^36^. In this context, the greater glucose transport of transformed cells depends primarily on the upregulation of *SLC2A1* (glucose transporter 1, *GLUT1*), responsible for basal glucose transport in all cell types^37^, and, herein, we propose a mechanism to explain how cancer cells can adapt to the extra requirements of glucose: SLC2A1 overexpression mediated by the epigenetic silencing of *DERL3*, the gene responsible for its degradation. The finding that *DERL3* hypermethylation occurs across a wide spectrum of tumour types is also a likely cause of the observed high level of expression of SLC2A1 in human primary tumours that is correlated with invasiveness and metastatic potential^26^.

The increased tumorigenesis potential of cells with *DERL3* epigenetic silencing, and thus overexpression of SLC2A1, could also be related to the high levels of secreted lactate that they produce. Thus, it has been suggested that more than being merely a waste product, lactate actively promotes tumour invasiveness and angiogenesis^38^. In this regard, the inability of the immune system to eliminate cancer cells might be related to the elimination of infiltrated lymphocytes by the aberrant lactate concentration in the tumour milieu^39^. It is interesting to note that for colorectal tumours, the original cancer type in which we first identified *DERL3* epigenetic silencing and for which there is only one report of aberrant DNA methylation of its promoter^40^, the lactate derived from microbiota is also known to increase the overall number of aberrant crypt foci^41^.

One of the most exciting possibilities arising from the recognition that cancer cells depend on glycolysis^19^,^20^,^21^ is the use of inhibitors of the various glycolytic enzymes, such as hexokinase, lactate dehydrogenase, ATP citrate lyase, phosphoglycerate mutase and pyruvate kinase as anticancer agents^22^,^23^. Herein, we have observed that SLC2A1-overexpressing cells, due to *DERL3* transcriptional inactivation, are sensitive to inhibitors of the M2 isoform of pyruvate kinase (PKM2). PKM2 is a glycolytic enzyme that catalyses the production of pyruvate and ATP from phosphoenolpyruvate and adenosine 5′-diphosphate^33^. PKM2 promotes aerobic glycolysis and tumour growth *in vivo*, while its inhibition blocks cell proliferation^42^,^43^. The question then arises about how the DERL3-mediated upregulation of SLC2A1 can be mechanistically linked to the observed higher sensitivity to PKM2 inhibitors. The answer could be provided by the function of PKM2 as a transcriptional coactivator^44^,^45^,^46^ that promotes the upregulation of SLC2A1 (ref. 47). Thus, those cancer cells with extremely high levels of SLC2A1, such as those hypermethylated at DERL3, will be very sensitive to drugs that downregulate SLC2A1 expression, such as direct (shikonin) or indirect (AKT inhibitor VIII) PKM2 inhibitors. These findings might have significant consequences for the use of drugs that target glycolysis in personalized cancer therapy.

## Methods

### Cell lines and primary tumour samples

All the cell lines used in this study were purchased from the American Type Culture Collection (Rockville, MD), except HCT-116 and DKO, which were generous gifts from Dr Bert Vogelstein (Johns Hopkins Kimmel Comprehensive Cancer Center, Baltimore, MD, USA). Cells were cultured in DMEM medium supplemented with 10% fetal bovine serum at 37 °C and 5% CO_2_. Cell lines were treated with 5-aza-2′-deoxycytidine 1.5 μM for 72 h to promote DNA demethylation. DNA samples from primary tumours were obtained at the time of the clinically indicated surgical procedures. All patients provided informed consent and the study was conducted with the approval of the IDIBELL (Institutional Review Board of the Bellvitge Biomedical Research Institute).

### DNA methylation analyses

CpG islands were identified *in silico* using Methyl Primer Express v1.0 (Applied Biosystems, Carlsbad, CA, USA). DNA methylation profiles were obtained by bisulphite genomic sequencing of at least eight clones and methylation-specific PCR with primers specific for methylated or unmethylated DNA. In both cases, genomic DNA was first modified with bisulphite-mediated conversion of unmethylated cytosines, but not methylated, to uracil and purified using the EZ DNA Methylation-Gold Kit (ZYMO Research). Primers are listed in Supplementary Table 5. The DNA methylation microarray from Illumina (Infinium HumanMethylation450 BeadChip) was also used as previously described^16^,^48^. A three-step normalization procedure was performed using the lumi (package available for Bioconductor, within the R statistical environment), consisting of colour bias adjustment, background level adjustment and quantile normalization across arrays. Raw intensity DNA methylation files of an additional cohort of primary samples were obtained from The Cancer Genome Atlas data portal (https://tcga-data.nci.nih.gov/tcga/). The methylation level (β-value) for each of the 485,577 CpG sites was calculated as the ratio of methylated signal divided to the sum of methylated and unmethylated signals plus 100. DNA methylation analysis of *DERL3* was performed using CpG sites located in the CpG island and close proximity to the gene transcription start site. For primary samples, an average promoter methylation >0.33 was considered as hypermethylated and <0.25 as hypomethylated. For cancer cell lines, we required the hypermethylated promoter to display CpG methylation levels >75% in at least 60% of the analysed CpG sites.

### RNA extraction and real-time PCR

Total RNA was extracted from cell lysates using TRIzol Reagent (Invitrogen), purified using the RNeasy Kit (Qiagen) and 2 μg were retrotranscribed using the ThermoScriptTM RT-PCR System (Invitrogen). Real-time PCR reactions were performed in triplicate on an Applied Biosystems 7,900HT Fast Real-Time PCR system using 20 ng cDNA, 5 μl SYBR Green PCR Master Mix (Applied Biosystem) and 150 nM specific primers (detailed in Supplementary Table 5) in a final volume of 10 μl. To normalize the data, we used the *B2M* gene as an endogenous control.

### *DERL3* cloning in pIRES2-eGFP vector and generation of stable cell lines

Primers for the *DERL3* gene transcript were designed to clone it into the pIRES2-eGFP vector (BD Biosciences Clontech). Primers were designed to contain a FLAG-Tag in the carboxy-terminal end of the protein separated by a flexible Gly-Ser-Gly sequence (primers used are presented in Supplementary Table 5). 5 × 10^6^ HCT-116 cells were transfected by electroporation using 10 μg of each vector. Seventy-two hours later, cells were diluted and treated with G418 (Seralab) to force individual clone formation. Twenty-four clones were selected to analyse DERL3 recovery. Cell pellets were separated by SDS–PAGE, transferred to 0.22-μm nitrocellulose membranes (Whatman, GE Healthcare) and hybridized with anti-FLAG horseradish peroxidase (HRP)-conjugated antibody (M2, Sigma). Positive clones were then purified by cell sorting (MoFloTMXDP, Beckman Coulter). The proper positioning of DERL3 in the ER was checked by immunofluorescence. Cells were cultured directly on coverslips and fixed with 4% paraformaldehyde in PBS for 30 min at room temperature. Cells were permeabilized with 0.1% Triton X-100 in PBS for 10 min at room temperature and blocked with 2% blocking reagent (Roche) for 1 h. Immunostaining with anti-FLAG-M2-Cy3 (1:500) (Sigma) and anti-Calnexin (1:1,000) (ab31290, Abcam) was performed for 16 h at 4 °C. The coverslips were mounted on glass slides with Mowiol and 4′,6-diamidino-2-phenylindole. Multi-colour immunofluorescence images were then analysed under a Leica SP5 laser scanning confocal spectral microscope (Leica Microsystems, Germany) equipped with Argon, DPSS561, HeNe633 and 405 Diode and a × 63 oil-immersion objective lens (NA 1.32).

### Cell proliferation and drug dose-response assays

Cell viability and proliferation were determined by the MTT assay. A total of 800 cells were plated onto 96-well microdilution plates and MTT added on 7 consecutive days at a final concentration of 5 mg ml^−1^. After incubation at 37 °C and 5% CO_2_ for 3 h, the MTT was removed and MTT formazan crystals were dissolved in 100 μl of DMSO (dimethyl sulphoxide). Absorbance at 570 nm was determined on an automatized microtiter plate reader (BioRad). For colony formation experiments, colonies of cells were fixed and stained with crystal violet reagent. For dose-response assays, 800 cells were plated onto 96-well microdilution plates. Following overnight cell adherence, experimental medium containing the appropriate drug concentration or control media was added to the wells. After 3 days, the MTT assay was carried out, as described previously.

### Mouse xenograft and metastasis models

Athymic nude male mice were subcutaneously injected in each flank with a total of 3.5 × 10^6^ empty vector cells (right flank) or DERL3-transfected HCT-116 cells (left flank) soaked in Matrigel (BD Biosciences). Tumour growth was monitored every 2 days by measuring tumour width (*W*) and length (*L*). Tumour volume, *V*, was then estimated from the formula *V*=*π*/(6 × *L* × *W*^2^). Mice were killed 32 days after injection and tumour weight measured.

For the metastasis model, 1.5 × 10^6^ cells were injected into the spleen of 20 mice (10 mice for each condition). To avoid local tumour growth, the spleens were removed 48 h after cell injection. All the empty vector mice were killed 5 weeks later and the hepatic metastases examined. Equal numbers of the DERL3-transfected mice were killed 5 and 9 weeks after cell injection to examine early and late hepatic metastases, respectively. Hepatic metastases were examined macroscopically and microscopically following hematoxylin and eosin stain tissue staining. For the drug treatment experiments, 3.5 × 10^6^ empty vector and DERL3-transfected HCT-116 cells were subcutaneously injected in nude mice. When the tumour volumes reached a volume of ~300 mm^3^, either shikonin (Sigma) (2 mg kg^−1^ dissolved in saline buffer with 10% DMSO) or carrier solution (saline buffer with 10% DMSO) was injected into the peritoneal cavity every other day for 1 week. All experiments were approved by the IDIBELL Animal Care and Use Committee.

### SILAC labelling and protein analysis

SILAC labelling was performed as previously described^49^. Briefly, DMEM medium without lysine and arginine (Silantes) was supplemented with 10% dialysed fetal bovine serum (Silantes), 300 mg l^−1^ proline (Sigma) and either 0.8 mM lysine plus 0.4 mM arginine (both from Sigma) for the light condition or 0.8 mM ^13^C_6_-lysine plus 0.4 mM ^13^C_6_-arginine (both from Silantes) for the heavy condition. DERL3 and empty vector-transfected HCT-116 cells were grown in light and heavy medium, respectively, for 7 days. After labelling, cells were counted and mixed in a 1:1 ratio, washed twice with PBS and stored at −70 °C until further processing. Pellets containing about 2 × 10^6^ cells were resuspended in 200 μl of lysis buffer (1% Igepal CA-630, 10 mM Tris-HCl, 150 mM NaCl, 0.02% NaN_3_, 1 mM EDTA) with a mixture of protease inhibitors (Complete Mini without EDTA, Roche) and incubated at 4 °C for 30 min with gentle shaking. The lysate was centrifuged at 16,000 *g* for 10 min and proteins in the soluble fraction were extracted by methanol/chloroform precipitation. In other experiments, about 2 × 10^6^ cells were lysed and fractionated using a commercially available protein extraction kit (Proteoextract, subcellular proteome extraction kit, Calbiochem) following manufacturer's instructions. In this case, only the membrane/organelle fraction was used for further analysis. Proteins were fractionated in 12% SDS–polyacrylamide minigels. After staining with Page Blue (Thermo), the gel was cut into 12 bands that were trypsin digested in a Proteineer DP digest robot (Bruker Daltonics).

### Liquid chromatography–mass spectrometry/mass spectrometry analysis

The tryptic peptide pool was analysed in a nano-LC Ultra HPLC (Eksigent) coupled online with a 5,600 triple time of flight mass spectrometer (AB Sciex). Mass spectrometry/mass spectrometry spectra were matched to putative peptide candidates in a target/decoy version of the Uniprot Knowledgebase database using three search engines: MASCOT (from Matrix Science) v2.4, OMSSA v2.1.9 and TANDEM 13-02-01-1. Cysteine carbamidomethylation was set as fixed modification, while methionine oxidation, pyroglutamic acid from peptide N-terminal glutamine or glutamic acid, acetylation at protein N terminus and SILAC labels in lysine or arginine residues were set as variable. Tolerance values of 0.01 Da and 0.02 Da were used for parent and fragment ions, respectively, assuming fully tryptic cleavage specificity and allowing for up to two missed cleavage sites. Quadrupole time-of-flight (QTOF)-specific scoring was enabled in MASCOT searches. Search results were filtered as described elsewhere^50^, and merged at a peptide-level FDR of <0.01. Identified peptides were clustered in redundancy groups, each group including all hits from proteins sharing at least one identified peptide. A data-processing workflow from Proteobotics (Madrid, Spain) was used to locate SILAC signals of each protein group in raw spectra. Alignment, noise removal, signal fitting and filtering were carried out according to default automatic procedures. High-quality signals were used to generate log ratios and fit the built-in nonlinear mixed model. *P*-values were assigned to each average *n*-fold change group by generating 10^5^ random draws from the fitted model. Statistical significance for differential protein expression was assessed by using FDR values computed from the *P*-values that consider the number and quality of measured signals simultaneously for the degree of replication used.

### Western blot and cycloheximide chase assay

Membrane proteins were isolated by subcellular fractioning using the ProteoExtract Subcellular Proteome Extraction Kit (Calbiochem). Protein concentration was determined by Bradford (BioRad). DERL3 levels were analysed by western blot using polyclonal anti-DERL3 antibody (1:500) (ab78233, Abcam). SLC2A1 (glucose transporter 1, GLUT1) levels were analysed by western blot, cell cytometry and immunofluorescence using polyclonal anti-SLC2A1 antibody (1:2,000) (ab652, Abcam) and anti-rabbit IgG conjugated to HRP (GE Healthcare) as the secondary antibody. Total protein extracts and anti-ubiquitin antibody (1:1,000) (MAB1510, Millipore) were used to determine global ubiquitin levels. For the cycloheximide chase assay, *DERL3* and empty vector HCT-116 stably transfected cells were transiently transfected with pcDNA4/TO vector (Invitrogen) containing the *SLC2A1* transcript fused to HA-tag and pcDNA3.1 (+) containing *VCP* fused to RGS-His-tag. Forty-eight hours later, protein synthesis was inhibited by supplying cycloheximide (Sigma) to the culture medium at a final concentration of 100 μg ml^−1^. Samples were recovered every 12 h and target degradation analysed by western blot using the anti-tag antibodies: anti-FLAG conjugated to HRP (1:1,000) (2M, Sigma), anti-HA (1:1,000) (Sigma) and anti-RGS-His (1:1,000) (Qiagen). Anti-ACTB antibody conjugated to HRP (1:10,000) (Sigma) was used as a loading control. Uncropped scans of western blots are included in Supplementary Fig. 12.

### Glucose uptake measurement

Cells were cultured in 24-well dishes and grown to 50% confluence. Cells were washed and medium without glucose was added in the presence of 100 μM 2-deoxy-D-glucose (Sigma) and 2-deoxy-D-[1,2-3H]glucose (Perkin-Elmer Ref. NET 328001, 8.5 Ci mmol^−1^, 1 μCi ml^−1^). Cells were incubated at 37 °C for 5 or 10 min. 2-deoxy-D-glucose uptake was stopped by the addition of 1 ml of ice-cold 50 mM glucose in PBS. Cells were washed three times in the same solution and disrupted with 0.1 M NaOH/0.1% SDS. Radioactivity was determined by scintillation counting. Protein was determined by the Bradford method. Each condition was run five times.

### Lactate production measurement

Cells were cultured in glucose-, pyruvate- and glutamine-free media (Gibco) supplemented with 10% dialysed fetal bovine serum (Gibco) and 4, 5 or 1 g l^−1^ of glucose for high- or low-glucose conditions. Supernatant samples were recovered every 24 h for 3 days and lactate secretion was quantified using an enzymatic reaction based on the oxidation of L-lactate to pyruvate by lactate dehydrogenase (Roche) in the presence of oxidized nicotinamide adenine dinucleotide (NAD) (Sigma-Aldrich). The amount of reduced nicotinamide adenine dinucleotide (NADH) produced in the reaction is proportional to the amount of L-lactate in the samples. NADH concentration was determined by measuring absorbance at 340 nM.

### Measurement of oxygen consumption

HCT-116 DERL3-transfected and DERL3-untransfected cells were detached with HyQ protease (HyClone) and resuspended in HBS (Hepes-buffered saline) at a final concentration of 1–2 × 10^6^ cells ml^−1^. Oxygen consumption was measured using a high-resolution oxygraph (Oxygraph-2K; Oroboros Instruments, Innsbruck, Austria). Respiratory activity was calculated as the time derivative of oxygen concentration measured in the closed respirometer and expressed per million viable cells. The amplified signal was analysed online to show the calibrated oxygen concentration and oxygen flux (DatLab software for data acquisition and analysis; Oroboros Instruments).

### ATP measurement

DERL3 and empty vector-transfected HCT-116 cells were recovered every 24 h for 3 days and intracellular ATP levels were quantified using the ENTILEN ATP Assay (Promega) according to the manufacturer’s instructions. Briefly, cell pellets were suspended in 200 μl of physiologic serum and ATP extracted with ice-cold 2.5% trichloroacetic acid. Trichloroacetic acid was neutralized and diluted to a final concentration of 0.1% by adding 1M Tris-acetate pH 7.75. Samples were centrifuged at 10,000 *g* for 5 min and supernatant used for ATP measurement. Luminescence was measured in a GloMax-Multi+ Detection System (Promega). Results were normalized to cell number.

### Measurement of mitochondrial ROS production

To detect the live-cell mitochondrial ROS we used the Mitosox red probe (Msox, Invitrogen, Carlsbad, CA, USA). HCT-116 DERL3-transfected and -untransfected cells were incubated with Msox to a final concentration of 5 μM for 10 min at 37 °C in the dark. Cells were then washed with Hank's balanced salt solution (HBSS), and Msox red fluorescence was measured with a FluoSTAR OPTIMA fluorescence plate reader (BMG Labtech).

### Measurement of ROS generation

The oxidation-sensitive fluorescent probe 2′,7′-dichlorodihydro-fluorescein diacetate (H_2_DCFDA) (from Invitrogen) was used to analyse the total intracellular content of ROS. HCT-116 DERL3-transfected and -untransfected cells were incubated with 2.5 μM H_2_DCFDA (30 min at 37 °C in the dark) in HBSS without red phenol. They were then lysed for 10 min at 4 °C with lysis buffer (25 mM HEPES pH 7.5, 60 mM NaCl, 1.5 mM MgCl_2_ and 0.1% Triton X-100) and transferred in duplicate to a 96-well plate. Fluorescence of the DCF subproduct was measured with a FluoSTAR OPTIMA fluorescence plate reader, and the results expressed as percentages of the control after correction for protein content (PierceBCA Protein Assay Kit, Thermo Scientific).

### SLC2A1 silencing by short hairpin RNA

HCT-116 cells were infected with lentivirus containing either *SLC2A1*-specific short hairpin RNA or scrambled short hairpin RNA in pGFP-C-shLenti plasmid (OriGene). Infected cells were then plated into 96-well microdilution plates to measure cell viability over 6 days by MTT assay as previously described.

## Author contributions

P.L.-S. performed all experiments. M.M., A.R.-F. and J.P.A. performed the proteomics work. A.V. and S.P. performed the mice experiments. A.P., L.L., J.E.W., F.S.-B., K.S., C.M., H.H. and J.S. assisted in the cellular and molecular studies. A.V. and X.S. performed the pathology analyses. A.M.-C., E.M.-B., J.B.B., T.F.Ø., C.L.A. and J.T. analysed the clinical data. F.V. and J.C.P. assisted in the glycolysis experiments. U.M., M.B.B. and M.G.V.H. assisted in the drug sensitivity analyses. M.E. designed the study and wrote the manuscript.

## Additional information

**How to cite this article:** Lopez-Serra, P. *et al.* A *DERL3*-associated defect in the degradation of SLC2A1 mediates the Warburg effect. *Nat. Commun.* 5:3608 doi: 10.1038/ncomms4608 (2014).

## Supplementary Material

Supplementary Information Supplementary Figures 1-12 and Supplementary Tables 1-5

## Figures and Tables

**Table 1:** Number of mice showing peritoneal invasion and liver/lung metastasis.

	**HCT-116 empty vector-transfected cells**	**HCT-116 DERL3-transfected cells**	***P*****-value**
*Orthotopic implantation*			
*Peritoneal invasion*			
No	0	11	
Yes	12	1	<0.005
*Liver metastasis*			
No	2	10	
Yes	10	2	<0.01
*Lung metastasis*			
No	2	10	
Yes	10	2	<0.01
*Spleen injection*			
*Liver metastasis*			
No	4	8	
Yes	8	4	0.00734
*Lung metastasis*			
No	3	10	
Yes	9	2	<0.05

*P*-values obtained with the two-sample proportion test.
