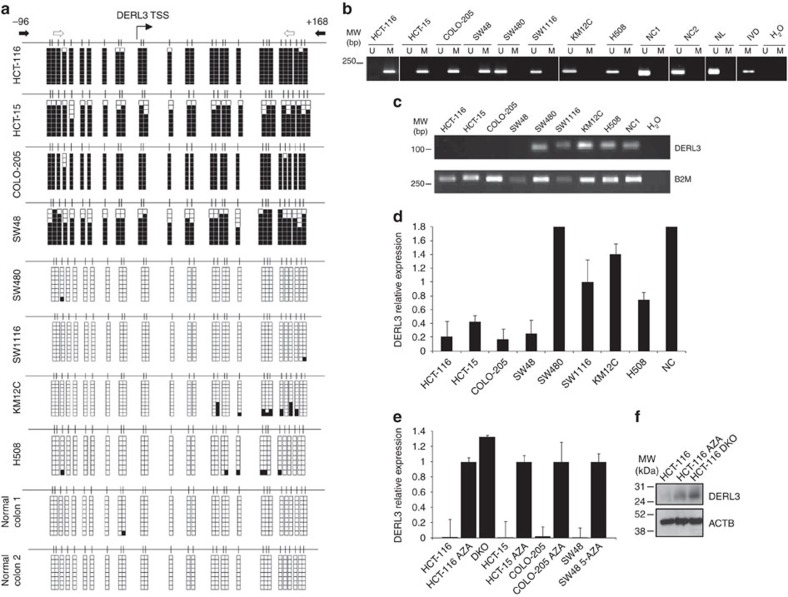# Corrigendum: A *DERL3*-associated defect in the degradation of SLC2A1 mediates the Warburg effect

**DOI:** 10.1038/ncomms13467

**Published:** 2016-11-02

**Authors:** Paula Lopez-Serra, Miguel Marcilla, Alberto Villanueva, Antonio Ramos-Fernandez, Anna Palau, Lucía Leal, Jessica E. Wahi, Fernando Setien-Baranda, Karolina Szczesna, Catia Moutinho, Anna Martinez-Cardus, Holger Heyn, Juan Sandoval, Sara Puertas, August Vidal, Xavier Sanjuan, Eva Martinez-Balibrea, Francesc Viñals, Jose C. Perales, Jesper B. Bramsem, Torben F. Ørntoft, Claus L. Andersen, Josep Tabernero, Ultan McDermott, Matthew B. Boxer, Matthew G. Vander Heiden, Juan Pablo Albar, Manel Esteller

Nature Communications 5: Article number: 4608; DOI: 10.1038/ncomms4608 (2014); Published: 04 03 2014; 11 02 2016

During the preparation of the PCR gel panels in Fig. 1b,c, duplicate or otherwise irrelevant lanes were excised; these excisions were not noted in the published figure as per the *Nature* journal policy. We are now correcting these figure panels as below. Figure 1b was assembled as a composite from multiple gels that were run in parallel using samples obtained from the same experiment. The corrected Fig. 1b below provides the demarcation as required by the *Nature* editorial policy. The original unprocessed gel images are provided in the Supplementary Information with the relevant corresponding bands denoted. Figure 1c was assembled from two gels run in parallel using samples obtained from the same experiment. We have not been able to provide all the original data used to assemble Fig. 1c. We have repeated the experiment and confirmed the original results; these new data are now reproduced below as the corrected Fig. 1c. The original unprocessed gel image is provided in the Supplementary Information. In addition, the original base pair markers in Fig. 1b were incorrect; the correct markers are shown below.

These corrections do not alter the original meaning of the experiments, their results, their interpretation, or the conclusions of the paper. We apologize for any confusion this may have caused to the readers of *Nature Communications*.

## Supplementary Material

Supplementary Information Supplementary Figures 1-6

## Figures and Tables

**Figure 1:**