# Supplementary material for: A DERL3-associated defect in the degradation of SLC2A1 mediates the Warburg effect
Source: Nat Commun. 2014 Apr 3;5:3608. doi: 10.1038/ncomms4608 (PMC3988805; doi:10.1038/ncomms4608)
Supplement: Supplementary Information — Supplementary Figures 1-12 and Supplementary Tables 1-5 [file ncomms4608-s1.pdf]

Supplementary Figure 1

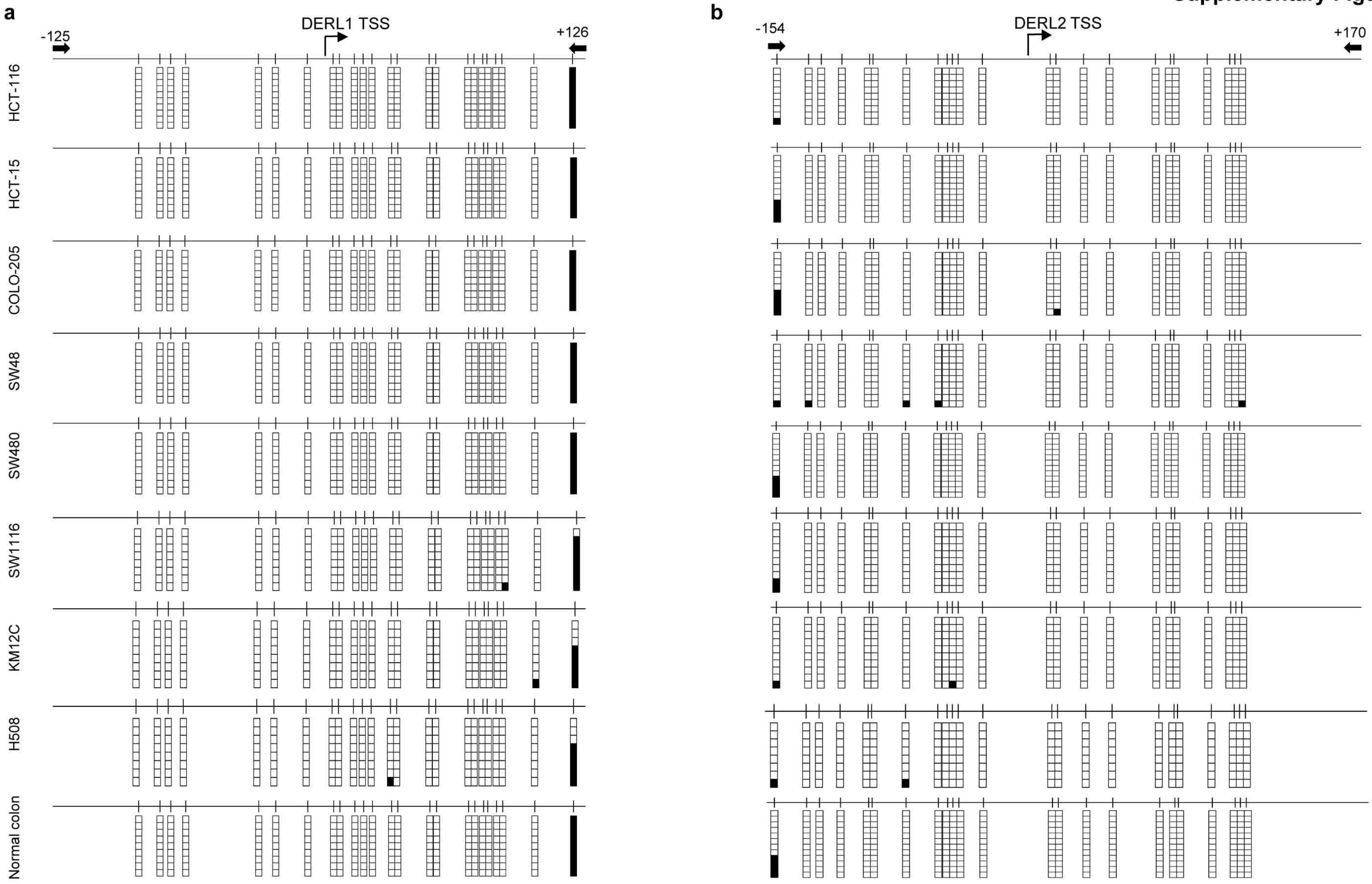

Supplementary Figure 1. *DERL1* and *DERL2* do not show DNA methylation on their gene promoter in colorectal cancer cell lines. *DERL1* (a) and *DERL2* (b) show an unmethylated CpG island in colorectal cancer cell lines. The vertical arrows represent the transcription start site (TSS) and the horizontal black arrows show the position of the bisulfite sequencing primers. White squares, unmethylated CpGs; balck squares, methylated CpGs.

	Probe ID	Chr.	Position	Density	Distance to TSS	Normal colon mucosa									
						NC01	NC02	NC03	NC04	NC05	NC06	NC07	NC08	NC09	NC10
DERL1	cg06528830	8	124054578	Island	-70	0.0	0.0	0.0	0.0	0.0	0.0	0.0	0.0	0.0	0.0
DERL1	cg12726672	8	124054668	Island	+20	0.0	0.1	0.1	0.1	0.1	0.1	0.1	0.1	0.1	0.1
DERL1	cg06932521	8	124054676	Island	+28	0.0	0.0	0.0	0.0	0.0	0.0	0.0	0.0	0.0	0.0
DERL1	cg06201372	8	124054732	Island	+84	0.1	0.1	0.1	0.1	0.1	0.1	0.1	0.1	0.1	0.1
DERL2	cg02550200	17	5389462	Island	-32	0.1	0.1	0.1	0.1	0.1	0.1	0.1	0.1	0.1	0.1
DERL2	cg07564061	17	5389550	Island	+56	0.0	0.0	0.1	0.0	0.0	0.0	0.0	0.0	0.0	0.0
DERL2	cg02222103	17	5389592	Island	+98	0.0	0.1	0.0	0.0	0.0	0.1	0.0	0.0	0.0	0.0
DERL2	cg21654974	17	5389607	Island	+113	0.0	0.0	0.0	0.0	0.0	0.0	0.0	0.0	0.0	0.0
DERL2	cg02842197	17	5389625	Island	+131	0.0	0.0	0.0	0.0	0.0	0.0	0.0	0.0	0.0	0.0
DERL2	cg07001481	17	5389669	Island	+175	0.0	0.0	0.0	0.0	0.0	0.0	0.0	0.0	0.0	0.0
DERL3	cg08369872	22	24181191	Island	-8	0.0	0.0	0.0	0.0	0.0	0.0	0.0	0.0	0.0	0.0
DERL3	cg11417653	22	24181212	Island	+13	0.0	0.0	0.0	0.1	0.0	0.1	0.0	0.0	0.0	0.0
DERL3	cg25940946	22	24181263	Island	+64	0.1	0.1	0.1	0.2	0.1	0.3	0.2	0.1	0.0	0.2
DERL3	cg25037461	22	24181268	Island	+69	0.1	0.1	0.2	0.2	0.1	0.2	0.2	0.1	0.1	0.1
DERL3	cg14774086	22	24181270	Island	+71	0.1	0.1	0.1	0.2	0.1	0.2	0.2	0.1	0.1	0.1

Probe ID	Colorectal cancer cell lines														
	SNU-C1	SW1417	LS-1034	CCK-81	SW837	HT55	RCM-1	HCC-56	SK-CO-1	HCC2998	NCI-H630	SW1116	CL-11	CW-2	T84
cg06528830	0.0	0.0	0.0	0.0	0.0	0.0	0.0	0.0	0.0	0.0	0.0	0.0	0.0	0.0	0.0
cg12726672	0.1	0.1	0.1	0.1	0.1	0.1	0.1	0.1	0.1	0.1	0.1	0.1	0.1	0.1	0.1
cg06932521	0.0	0.0	0.0	0.0	0.0	0.0	0.0	0.0	0.0	0.0	0.0	0.0	0.0	0.0	0.0
cg06201372	0.1	0.1	0.1	0.1	0.1	0.1	0.1	0.1	0.1	0.1	0.1	0.1	0.1	0.1	0.1
cg02550200	0.1	0.1	0.1	0.1	0.1	0.1	0.1	0.1	0.1	0.1	0.1	0.1	0.1	0.1	0.1
cg07564061	0.0	0.0	0.0	0.1	0.0	0.0	0.0	0.0	0.0	0.0	0.0	0.0	0.0	0.0	0.0
cg02222103	0.0	0.0	0.0	0.1	0.0	0.0	0.0	0.0	0.0	0.0	0.0	0.0	0.1	0.0	0.1
cg21654974	0.0	0.0	0.0	0.0	0.0	0.0	0.0	0.0	0.0	0.0	0.0	0.0	0.0	0.0	0.0
cg02842197	0.0	0.0	0.0	0.0	0.0	0.0	0.0	0.0	0.0	0.0	0.0	0.0	0.0	0.0	0.0
cg07001481	0.0	0.0	0.0	0.0	0.0	0.0	0.0	0.0	0.0	0.0	0.0	0.0	0.0	0.0	0.0
cg08369872	0.0	0.0	0.0	0.0	0.0	0.0	0.0	0.0	0.0	0.0	0.0	0.0	0.0	0.0	0.0
cg11417653	0.0	0.0	0.0	0.0	0.0	0.0	0.0	0.0	0.0	0.0	0.0	0.0	0.0	0.0	0.0
cg25940946	0.0	0.0	0.0	0.0	0.0	0.0	0.0	0.0	0.0	0.0	0.0	0.0	0.0	0.0	0.1
cg25037461	0.1	0.1	0.1	0.1	0.1	0.1	0.1	0.1	0.1	0.1	0.1	0.1	0.1	0.1	0.1
cg14774086	0.1	0.1	0.1	0.1	0.1	0.1	0.1	0.1	0.1	0.1	0.1	0.1	0.1	0.1	0.2

Probe ID	Colorectal cancer cell lines (continued)												
	C2BBE1	NCI-H716	LS-123	SW948	NCI-H508	CAR-1	SNU-C2B	SW1463	KM12	COLO-205	HCT-15	HCT-116	SW48
cg06528830	0.0	0.0	0.0	0.0	0.0	0.0	0.0	0.0	0.0	0.0	0.0	0.0	0.0
cg12726672	0.0	0.1	0.1	0.1	0.1	0.1	0.1	0.1	0.0	0.1	0.0	0.1	0.1
cg06932521	0.0	0.0	0.0	0.0	0.0	0.1	0.0	0.0	0.0	0.0	0.0	0.1	0.0
cg06201372	0.1	0.1	0.1	0.1	0.1	0.1	0.1	0.1	0.1	0.1	0.0	0.1	0.1
cg02550200	0.1	0.1	0.1	0.1	0.1	0.1	0.1	0.1	0.1	0.1	0.1	0.1	0.1
cg07564061	0.2	0.1	0.0	0.0	0.0	0.0	0.0	0.0	0.0	0.0	0.0	0.0	0.0
cg02222103	0.1	0.0	0.0	0.0	0.0	0.0	0.0	0.0	0.0	0.0	0.0	0.0	0.0
cg21654974	0.0	0.0	0.0	0.0	0.0	0.0	0.0	0.0	0.0	0.0	0.0	0.0	0.0
cg02842197	0.0	0.0	0.0	0.0	0.0	0.0	0.0	0.0	0.0	0.0	0.0	0.0	0.0
cg07001481	0.0	0.0	0.0	0.0	0.0	0.1	0.0	0.0	0.0	0.0	0.0	0.1	0.0
cg08369872	0.0	0.0	0.0	0.0	0.0	0.0	0.0	0.0	0.1	1.0	0.9	1.0	1.0
cg11417653	0.0	0.0	0.0	0.0	0.0	0.0	0.0	0.0	0.0	0.9	0.9	0.9	0.9
cg25940946	0.0	0.1	0.0	0.1	0.1	0.1	0.3	0.3	0.2	1.0	0.9	0.9	1.0
cg25037461	0.1	0.1	0.1	0.1	0.1	0.2	0.3	0.3	0.3	0.8	0.8	0.8	0.8
cg14774086	0.1	0.1	0.1	0.2	0.1	0.2	0.2	0.2	0.2	0.9	0.9	0.9	0.9

Supplementary Figure 2. The DNA methylation profile of the *DERL3* promoter is altered in colorectal cancer cell lines. Absolute DNA methylation levels (0-1) of the promoter-associated CpG islands of *DERL1*, *DERL2* and *DERL3* in 28 colorectal cancer cell lines and 10 normal primary colon tissue (NC). DNA methylation levels are color-coded (red: high, green: low). Probe distances to the transcription start site (TSS) are indicated.

Supplementary Figure 3

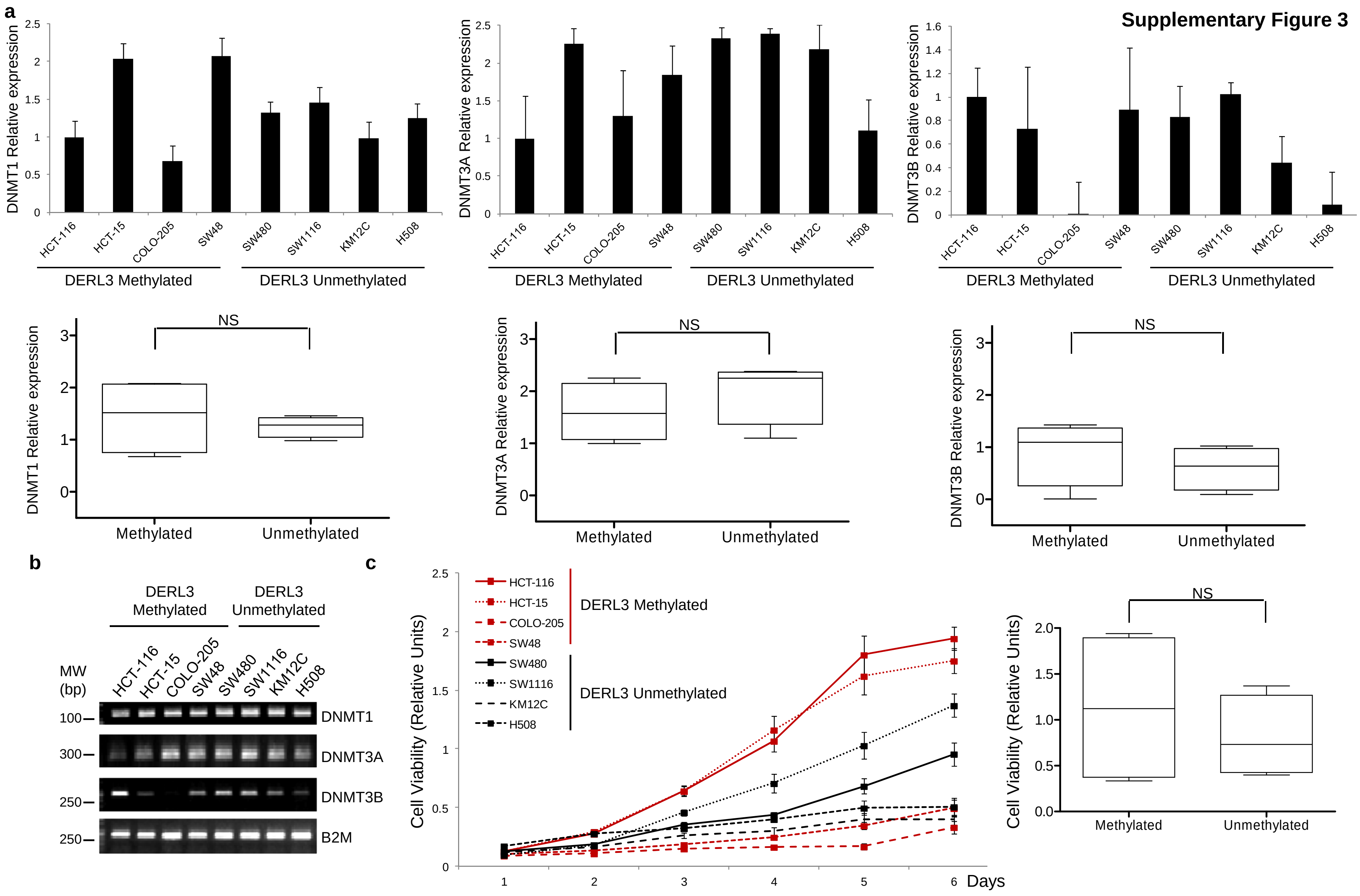

Supplementary Figure 3. (a) Top, DNA methyltransferases (*DNMT1*, *DNMT3A*, *DNMT3B*) gene expression in our panel of colorectal cancer cell lines analyzed by quantitative RT-PCR (data shown represent mean + SEM of biological triplicates); below, box plots of mean showing that there are no significant differences between the *DERL3* methylated and unmethylated groups (Mann-Whitney test). (b) semiquantitative PCR. *B2M*: beta-2-microglubulin. (c) MTT Cell viability assay in the panel of colorectal cancer cell lines. *DERL3* methylated cell lines (red); *DERL3* unmethylated cell lines (black). The box plots of mean show that there are no significant differences between *DERL3* methylated and unmethylated groups (Mann-Whitney test). NS: Non significant (p-value> 0.05).

Supplementary Figure 4

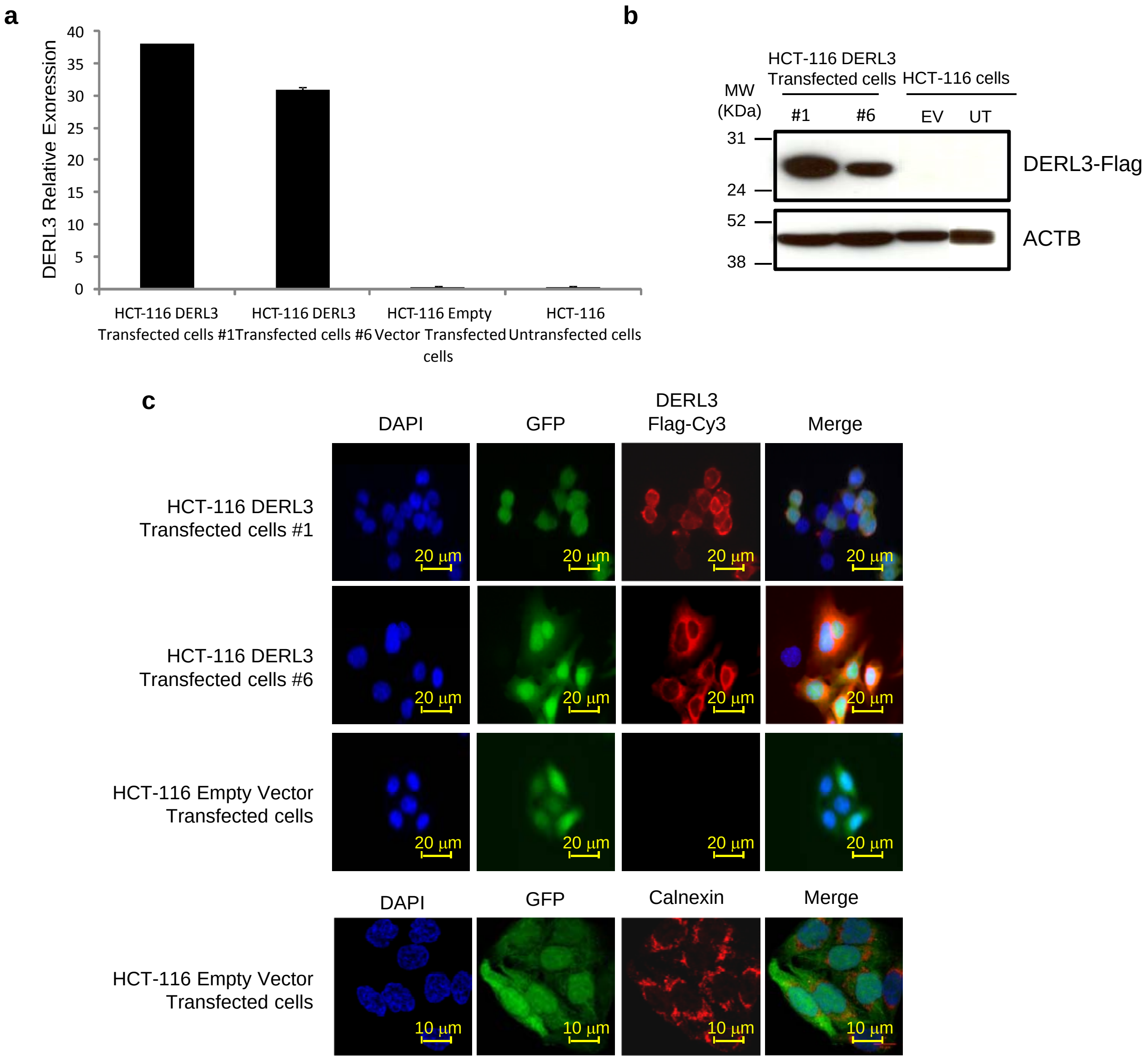

Supplementary Figure 4. **DERL3 expression recovery in HCT-116 cells**. (a) Full-length cDNA sequence of *DERL3* was cloned in pIRES2-eGFP plasmid for stable transfection in HCT-116 cells. Quantitative RT-PCR of two transfected clones (#1 and #6) shows the recovery of *DERL3* expression relative to HCT-116 empty vector transfected (EV) and untransfected (UT) cells. Data shown represent mean ± SEM (biological triplicates). (b) Western blot of DERL3-Flag demonstrates the efficient transfection at the protein level. EV: Empty vector; UT: Untransfected (c) Immunocytofluorescence studies of DERL3 and empty vector-transfected HCT-116 cells. Classic staining of the endoplasmic reticulum is showed by the Flag-Cy3 antibody, reflecting the correct location of DERL3 fused protein in the transfected cells. Calnexin hybridization was used as a positive control for endoplasmic reticulum staining.

Supplementary Figure 5

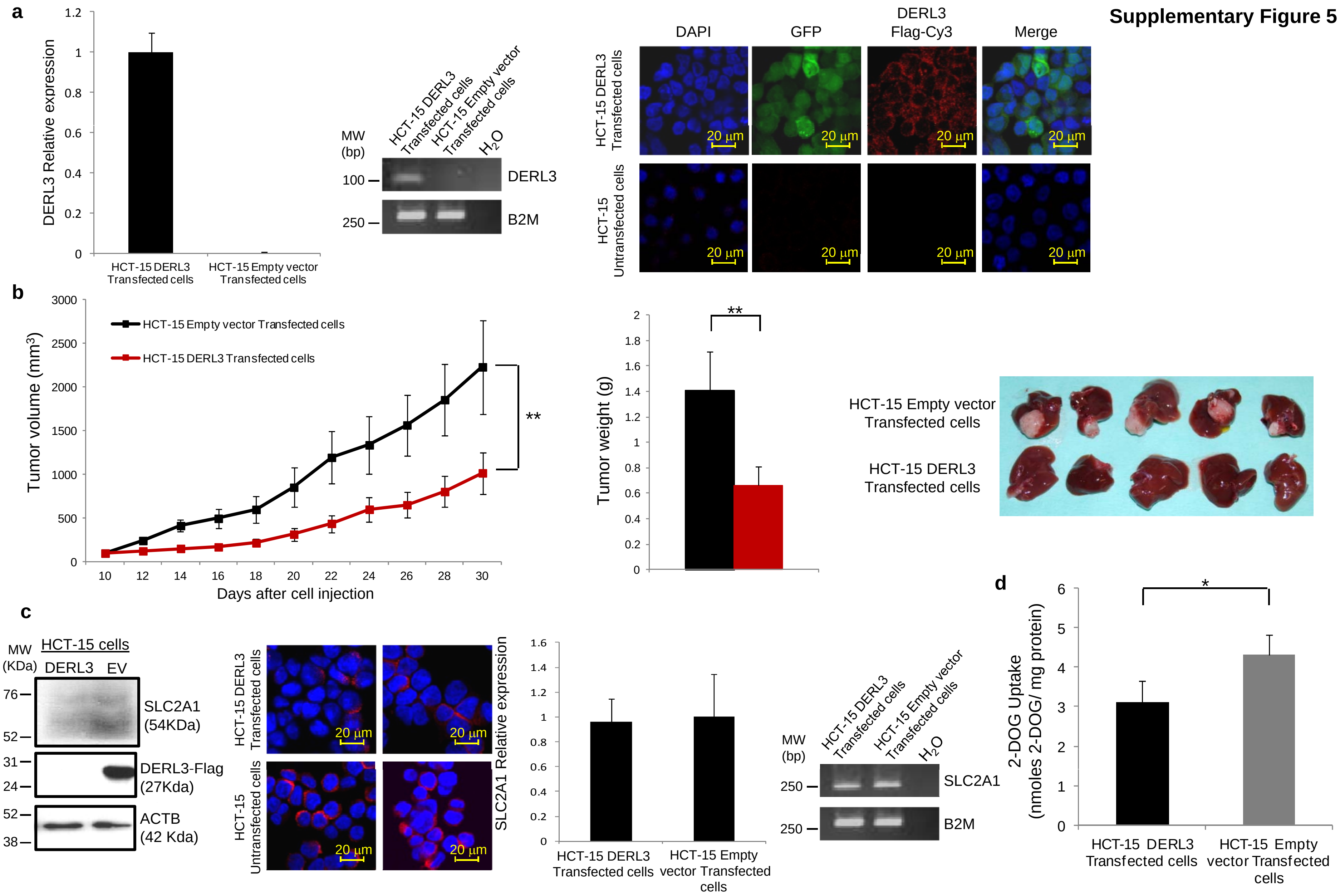

Supplementary Figure 5. Functional consequences of DERL3 recovery in HCT-15 cells. (a) Efficient restoration of *DERL3* in HCT-15 cells was confirmed by quantitative RT-PCR (data shown represent mean ± SEM of biological triplicates), semiquantitative PCR and immunofluorescence. (b) Subcutaneous tumors generated in nude mice showed a reduction in tumorigenic capability of HCT-15 DERL3 transfected cells (data shown represent mean ± SD) (Student´s t-test, p-val<0.01) , as well as a reduction in liver metastasis when the cells are injected directly in the spleen. (c) HCT-15 DERL3 transfected cells show reduced SLC2A1 protein levels as shown by western blot and immunofluorescence but not at messenger level, as shown by quantitative RT-PCR (data shown represent mean ± SEM of biological triplicates), and semiquantitative PCR. EV= Empty vector. (d) HCT-15 DERL3 transfected cells show a reduction in 2-DOG uptake (data shown represent mean ± SD) (Student´s t-test, p-val<0.05).

Supplementary Figure 6

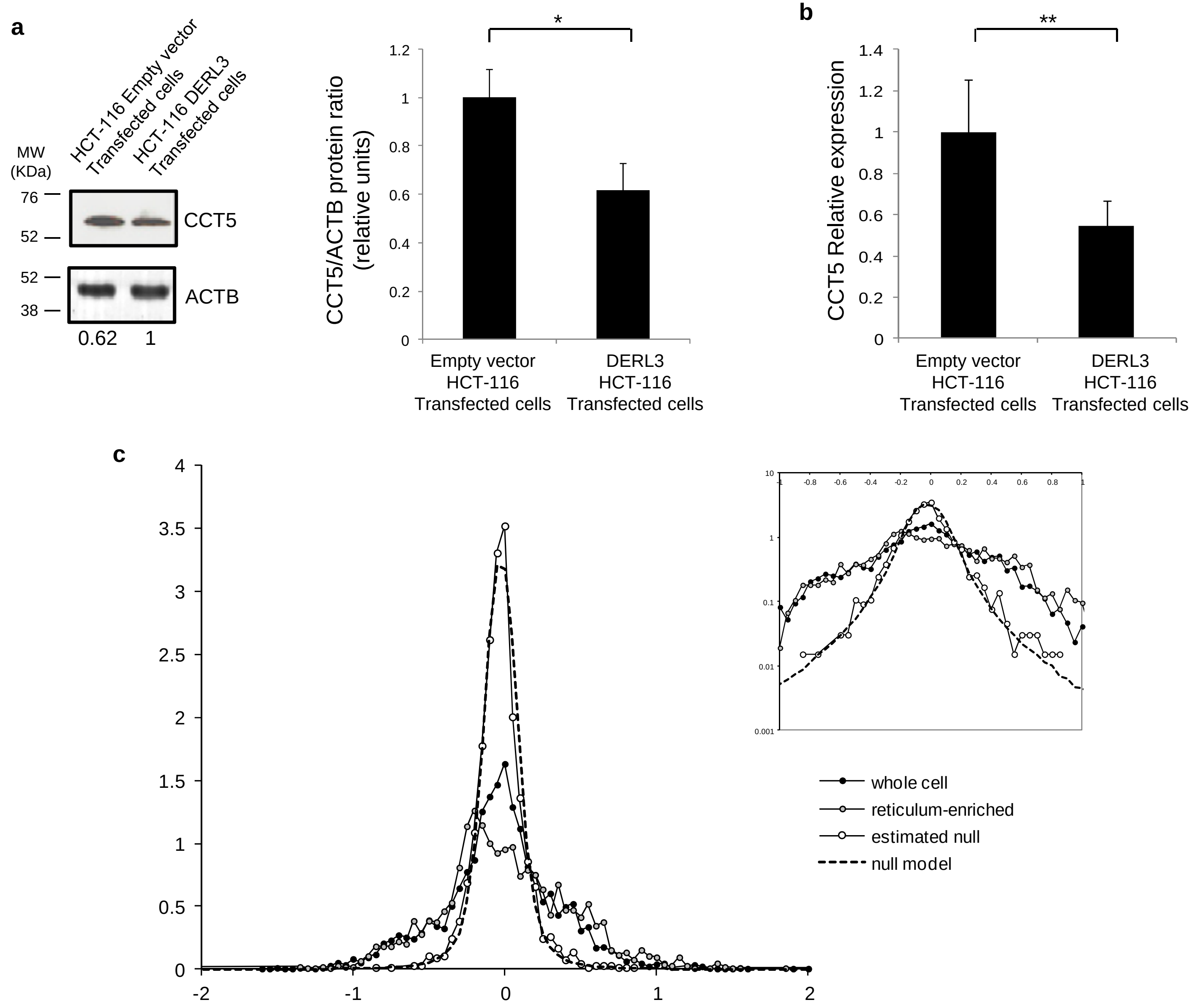

Supplementary Figure 6. (a) CCT5 protein is diminished in HCT-116 DERL3 transfected cells at the protein level as shown by the western blot (left panel). The right panel represents the quantification of the western blot bands (data shown represent mean ± SD) (Student´s t-test, p-val<0.05). (b) CCT5 downregulation is also observed at the RNA level, as shown by the quantitative RT-PCR (data shown represent mean ± SEM, biological triplicates) (Student´s t-test, p-val<0.01) . (c) log2 (fold-change) dispersion patterns of peptide signals in whole-cell lysate, reticulum-enriched fraction and non-differentially expressed proteins of both experiments, in decimal an logarithmic (upper right panel) scale.

Supplementary Figure 7

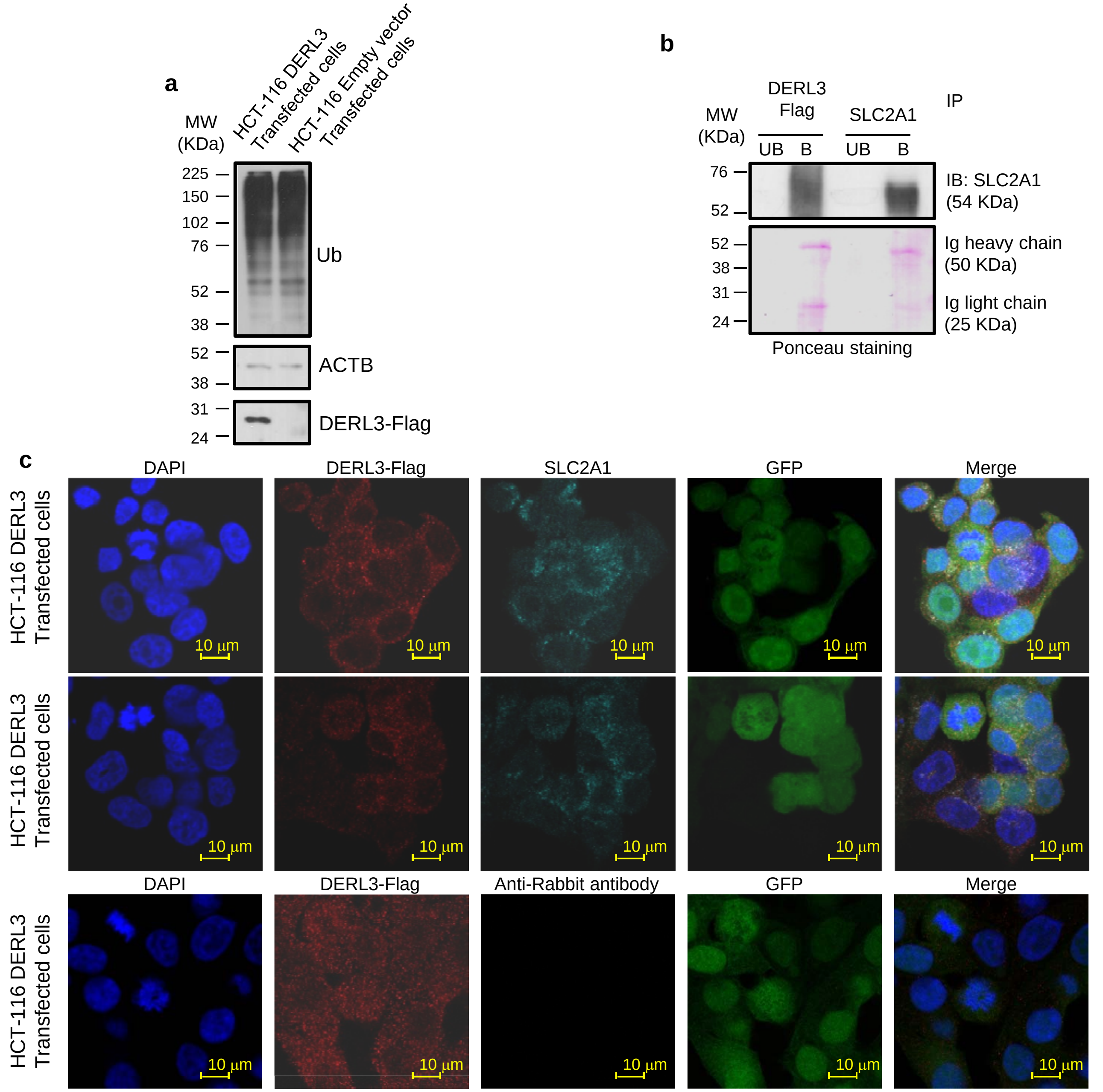

Supplementary Figure 7 (a) Western blot shows the absence of differences in global ubiquitin levels between DERL3-FLAG and pIRES2-eGFP HCT-116-transfected cells. (b) HCT-116 DERL3 Transfected cells were subjected to immunoprecipitation assay with anti-Flag antibody and anti SLC2A1 antibody; the western blot shows the interaction between DERL3 and SLC2A1 when DERL3 is pulled down (cropped image). The same conditions were used in both IPs, as shown by Ponceau staining. IP: immunoprecipitation; IB: immunoblotting; UB: unbound fraction; B: bound fraction. (c) DERL3 and SLC2A1 colocalize in the membrane of the endoplasmic reticulum (Mander´s overlap colocalization coefficient of 0.478).

Supplementary Figure 8

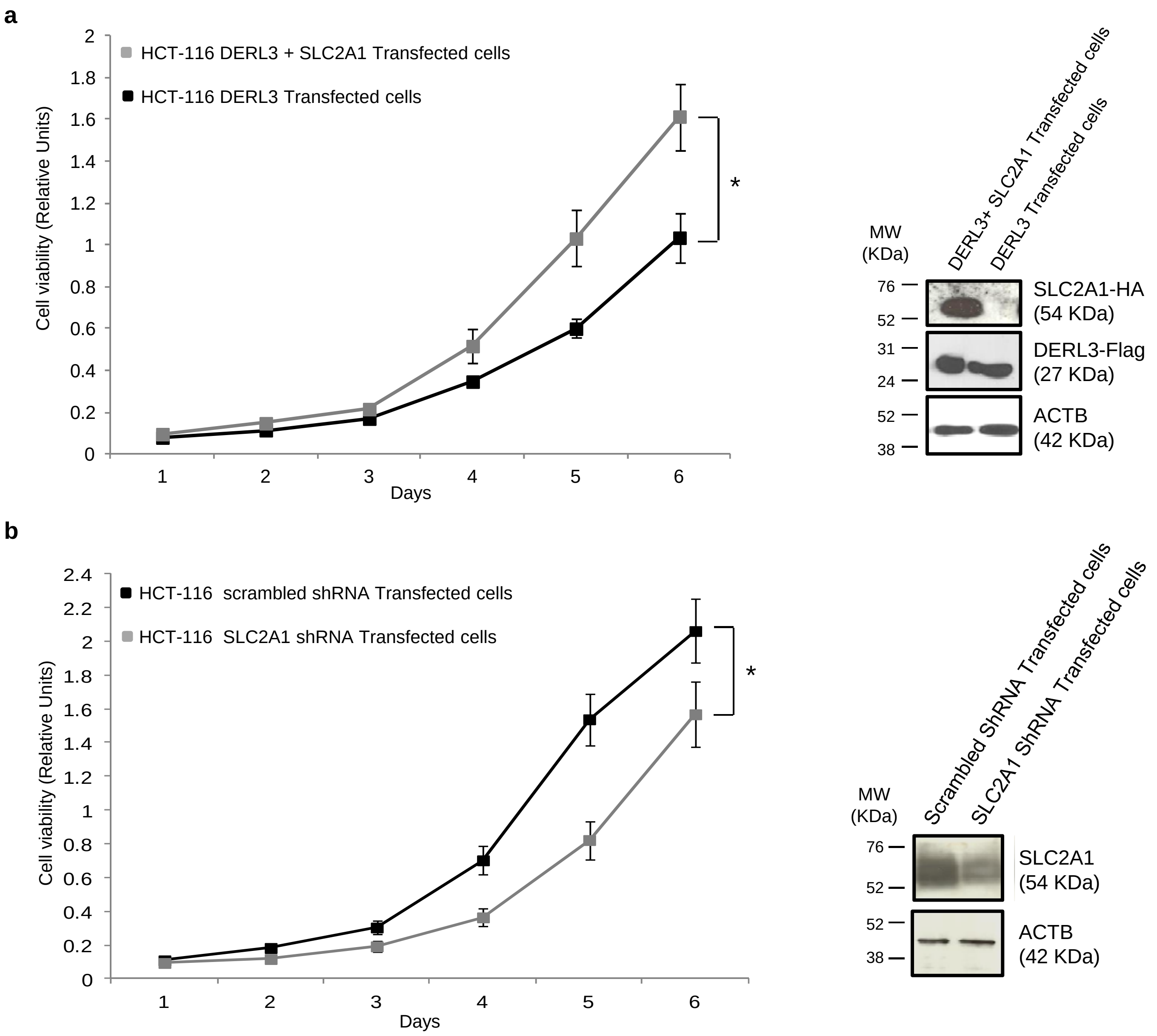

Supplementary Figure 8. (a) SLC2A1 recovery in HCT-116 DERL3 Transfected cells mitigates the effect of DERL3 overexpression in cell viability (data shown represent mean ± SD) (Student´s t-test, p-val<0.05). The right panel shows recovery of SLC2A1, HA tagged, by western blot. (b) SLC2A1 silencing by shRNA diminish the cell viability in HCT-116 (data shown represent mean ± SD) (Student´s t-test, p-val<0.05), the right panel show the downregulation of SLC2A1 upon shRNA transfection.

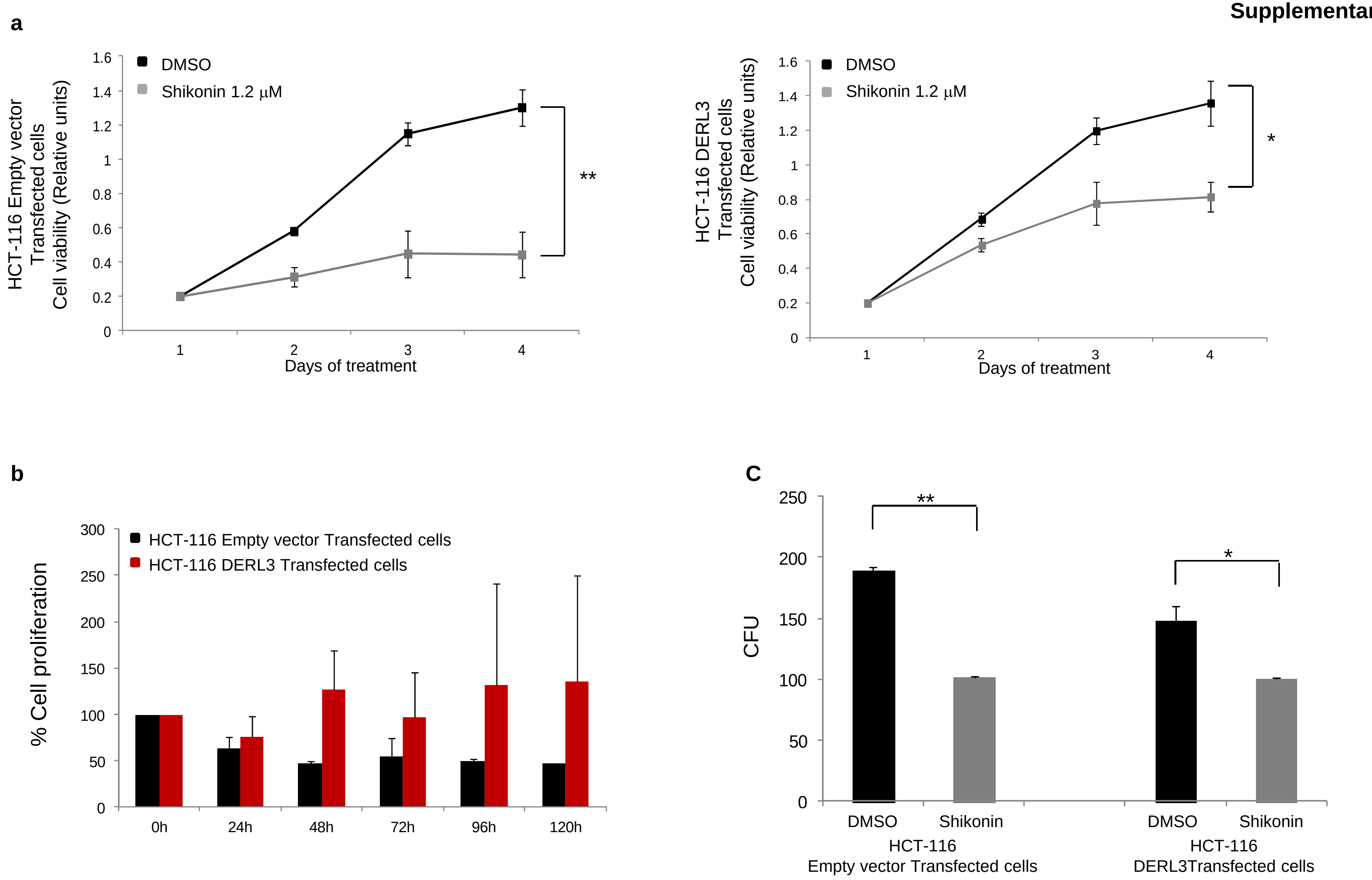

Supplementary Figure 9. HCT-116 cells show a higher sensibility to shikonin when compared to HCT-116 DERL3 transfected cells. (a) HCT-116 Empty vector cells showed a higher sensibility to shikonin treatment in a time-course experiment (data shown represent mean + SD) (Student´s t-test, **p-val<0.01, * p-val<0.05). SRB Cell proliferation assay (b) and colony formation assay (c) under shikonin treatment confirm the previous results (data shown represent mean + SD) (Student´s t-test, ** pval<0.01, * p-val<0.05). CFU: Colony Formation Units.

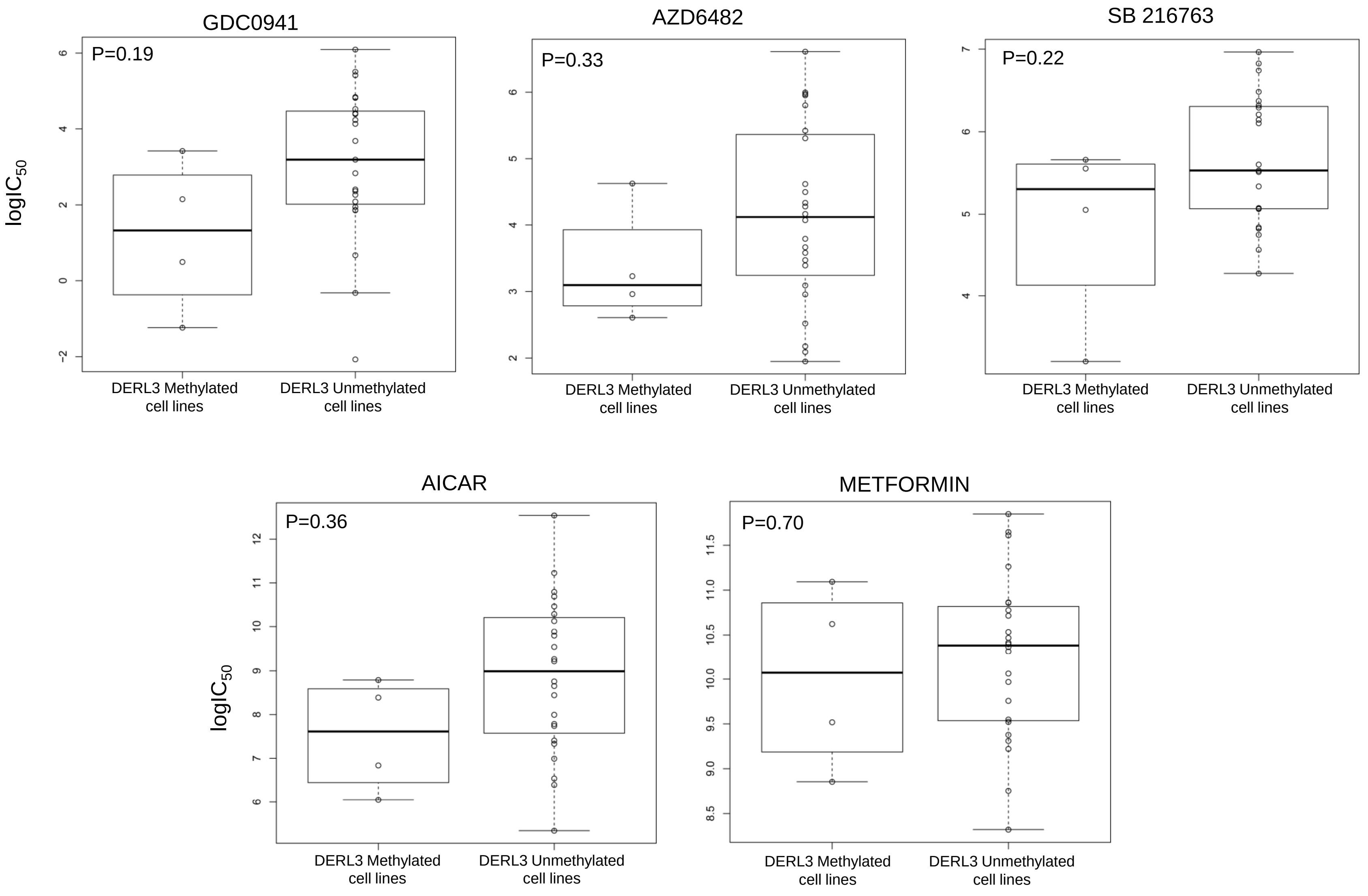

Supplementary Figure 10. DNA methylation levels of the *DERL3* promoter are associated to sensitivity to metabolic inhibitors. *DERL3* hypermethylated (n=4) and hypomethylated (n=22) colorectal cell lines displays differences in drug IC_{50} values (MANOVA test, corrected for multiple testing hypothesis) assessed by viability assays.

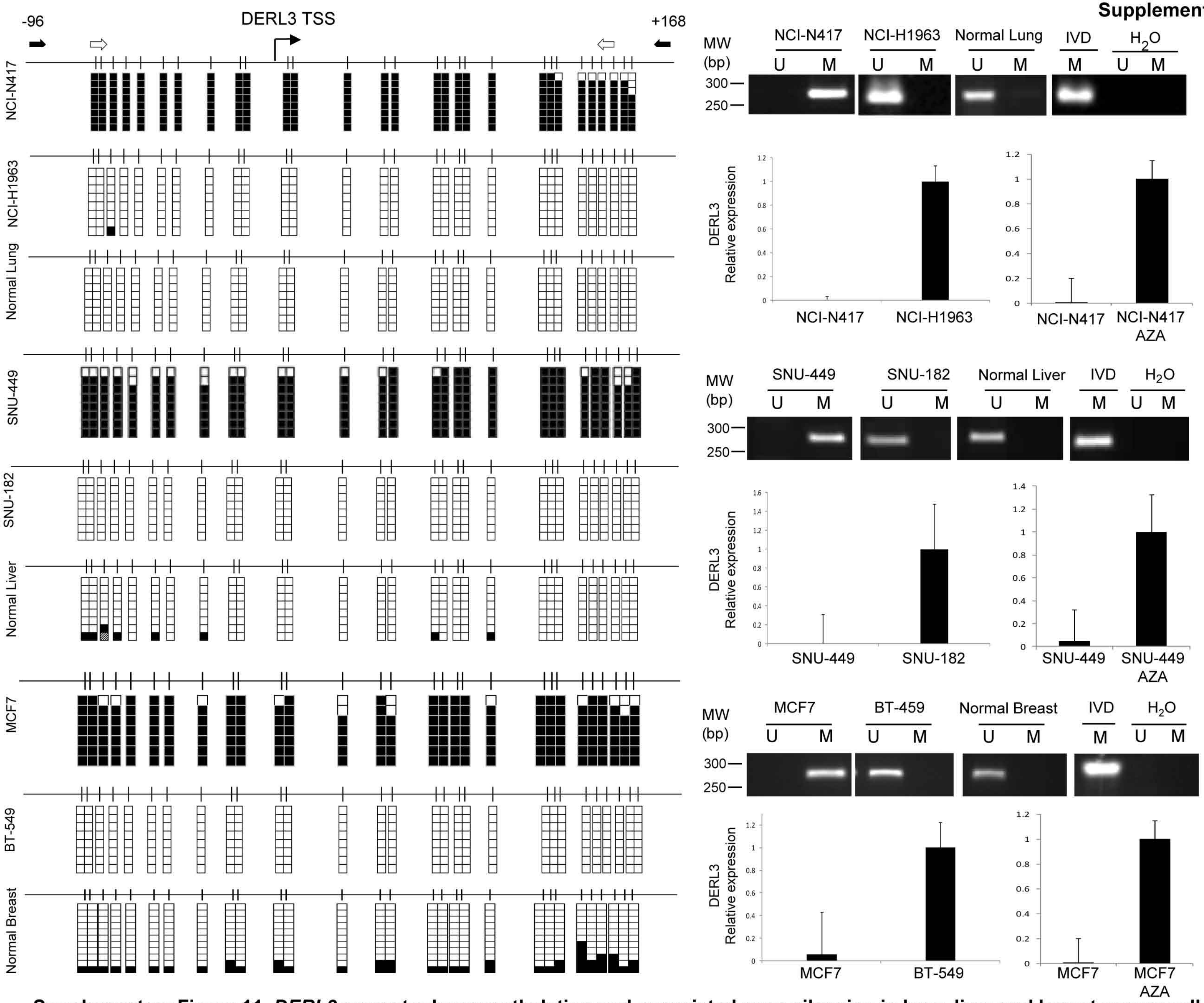

Supplementary Figure 11. ***DERL3* promoter hypermethylation and associated gene silencing in lung, liver and breast cancer cell lines.** Data shown represent mean ± SEM (biological triplicates).

Supplementary Figure 12

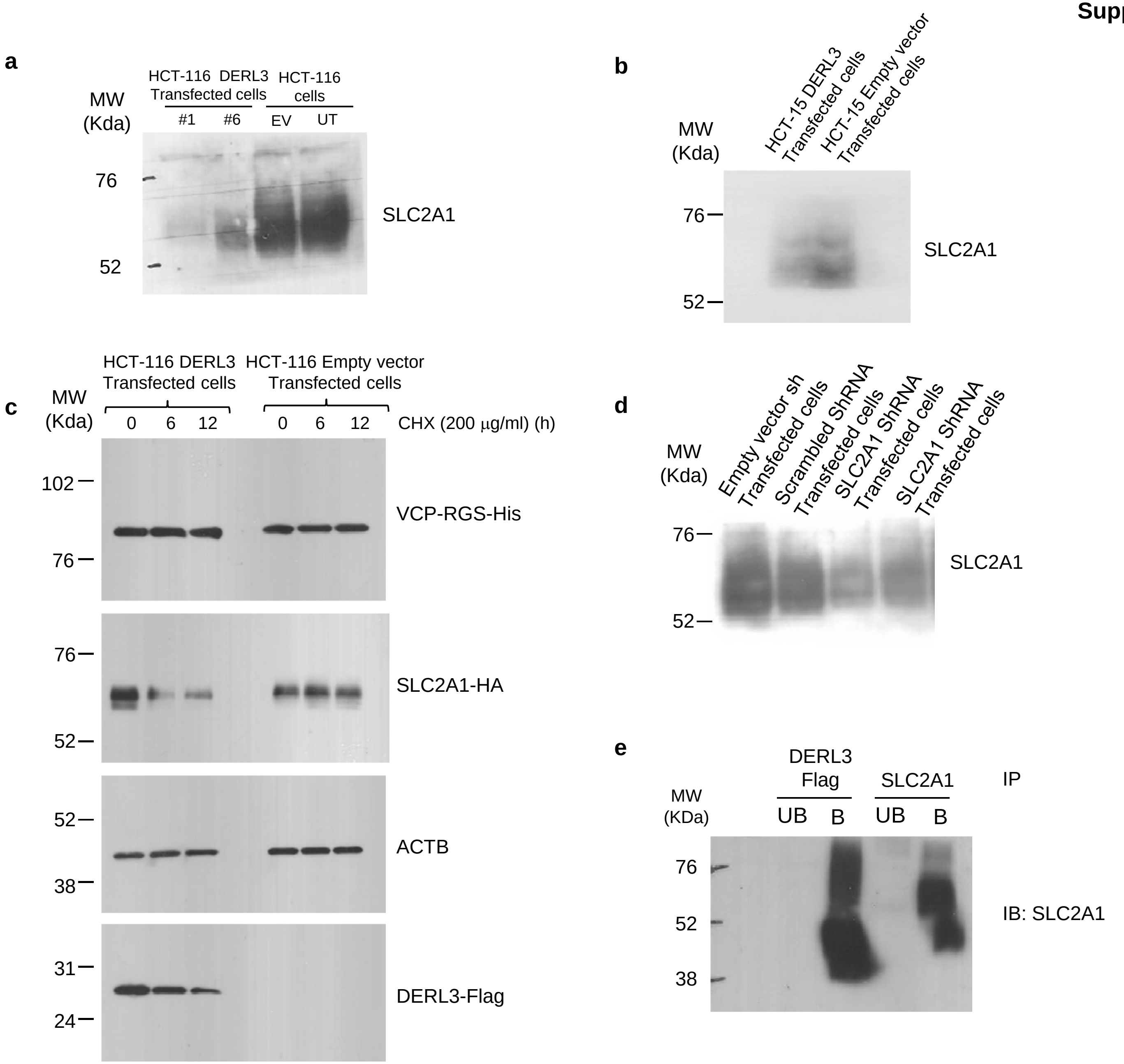

Supplementary Figure 12. Uncropped membranes of western blots. SLC2A1 downregulation upon DERL3 transfection in HCT-116 (a) and HCT-15 cells (b). (c) Cycloheximide chase assay showing SLC2A1 degradation in the presence of DERL3. (d) SLC2A1 downregulation upon shRNA transfection. (e) immunoprecipitation assay with anti-Flag antibody and anti SLC2A1 antibody for immunoblottiing.

Supplementary Table 1. List of thirty-three proteins represented for at least two peptides that underwent significant downregulation in the SILAC analyses upon *DERL3* transfection in HCT-116 cells.

contrast	p-value	FDR	ID Gene
-4.260761473	1.00E-05	0.000296906	SLC2A1
-1.471969136	3.00E-05	0.000544328	NQO1
-1.231432828	0.000489995	0.004495292	ALDH1A3
-1.095319801	0.000649994	0.005586523	PHGDH
-0.962521211	2.00E-05	0.000388806	CCT5
-0.956803262	1.00E-05	0.000291604	TCP1
-0.92198649	0.000199998	0.002299977	FAM3C
-0.856788698	2.00E-05	0.000429733	CCT6A, CCT6B
-0.837294153	1.00E-05	0.000709993	STMN1, STMN2
-0.822111093	1.00E-05	0.000340205	CCT8
-0.817685005	9.00E-05	0.001289198	TFRC
-0.812807356	0.001939981	0.011776908	TXNDC12
-0.789088543	1.00E-05	0.00068041	CCT3
-0.782825754	0.002199978	0.012876574	MPDU1
-0.775066717	0.002249978	0.013029125	CAPRIN1
-0.734052017	0.000139999	0.001800139	ANXA1
-0.718900767	1.00E-05	0.000408246	CROCC, RDX, EZR, MSN
-0.703742435	1.00E-05	0.000510307	CCT7
-0.68926798	0.000549995	0.004907874	EIF3I
-0.67557425	1.00E-04	0.001383884	HIST1H1
-0.674060605	9.00E-05	0.001300606	VARS
-0.665772802	0.003519965	0.016565137	PYCR2
-0.658645917	0.004669953	0.019553933	CKAP4
-0.646607536	2.00E-05	0.000403206	FLNB, FLNA
-0.603690884	1.00E-05	0.000251228	ATP1A
-0.602104541	0.003929961	0.017582537	ADRM1
-0.602033807	2.00E-05	0.000447393	SLC3A2
-0.596596479	0.004369956	0.018828862	TUBB
-0.555235714	0.005099949	0.020614398	ANXA2
-0.533891415	0.000569994	0.005004305	MAGEB2
-0.524520394	1.00E-05	0.000255154	TUBA
-0.520172565	0.002439976	0.013787129	EIF3
-0.514446208	3.00E-05	0.000569615	CCT3

Supplementary Table 2. List of twenty-seven proteins represented for at least two peptides that underwent significant upregulation in the SILAC analyses upon *DERL3* transfection in HCT-116 cells.

Fold-change	pvalue	FDR	IDsGene
**6.958586926**	**1.00E-05**	**0.000267702**	**DERL3**
3.857562434	1.00E-05	0.000907213	AHSG
2.432704206	3.00E-05	0.000550444	HSPH1
1.451480935	1.00E-05	0.000259204	PDIA3, PDIA3P
1.402771448	0.000209998	0.002301521	HRSP12
1.111380358	1.00E-05	0.000816492	ETFB
1.079300657	0.000809992	0.006646818	PTRH2
0.994102297	2.00E-05	0.000384231	SLC25A10;;MRPL12
0.978930383	1.00E-05	0.000286488	DECR1
0.960342402	0.001069989	0.007906301	BAX
0.872334856	0.000299997	0.003024044	AK2
0.852715115	0.000189998	0.002232136	TIMM50
0.851650165	1.00E-05	0.000388806	MDH2
0.794551603	1.00E-05	0.00204123	HSPE1
0.764874225	0.002129979	0.012602374	TOMM40
0.753399553	1.00E-05	0.001256141	FABP5
0.720246609	0.002919971	0.015283052	NOMO3, NOMO1, NOMO2
0.693801557	0.00297997	0.015448544	CLPTM1
0.691584012	1.00E-05	0.001632984	HSD17B10
0.673794805	0.003159968	0.015926631	SNRPA1
0.659222083	8.00E-05	0.001176925	TOMM22
0.652673233	7.00E-05	0.001039171	ATP5F1
0.599973901	0.004149959	0.018071686	PRKACA
0.552247262	0.001589984	0.010303349	ATP5H
0.549975585	0.001399986	0.009447013	CYCS
0.529525741	0.009739903	0.030295735	ATP6V1B1, ATP6V1B2
0.512936823	0.000259997	0.002704304	GSR

Supplementary Table 3. Frequency of *DERL3* Promoter CpG Island Hypermethylation in Cancer Cell Lines and Primary Tumors.

Sample Type	Cancer Cell Lines	Primary Tumors Current Study	Primary Tumors TCGA
Colorectal	4/24 (14.3%)	36/128 (28.1%)	69/250 (27.6%)
Acute Myeloid Leukemia	0/11 (0%)	0/73 (0%)	0/194 (0%)
Breast	16/30 (53.3%)	29/68 (42.6%)	284/588 (48.3%)
Bladder	1/15 (6.7%)	0/26 (0%)	8/167 (4.8%)
Endometrium	4/10 (40%)	4/43 (9.3%)	38/363 (10.5%)
Esophagus	19/28 (67.9%	6/15 (40%)	12/47 (25.5%)
Glioma	6/34 (17.6%)	8/44 (18.2%)	6/113 (5.3%)
Head and Neck	20/23 (87%)	4/13 (30.8%)	7/28 (25%)
Hepatocellular	5/8 (62.5%)	131/206 (63.6%)	42/105 (40%)
Kidney	0/23 (0%)	3/53 (5.7%)	35/459 (7.6)
Melanoma	3/34 (8.8%)	2/88 (2.3%)	33/314 (10.5%)
Non-Small Cell Lung	10/88 (11.4%)	35/504 (6.9%)	66/651 (10.1%)
Pancreas	5/25 (20%)	1/14 (7.1%)	2/65 (3.1%)
Prostate	1/5 (20%)	10/25 (40%)	60/168 (35.7%)
Thyroid	1/12 (8.3%)	0/14 (0%)	1/56 (1.8%)

TCGA, The Cancer Genome Atlas.

Supplementary Table 4. Frequency of *DERL3* Promoter CpG Island Hypermethylation in Normal Tissues (n=102)

Normal Sample Type	*DERL3* Hypermethylation	
Colorectal mucosa	0/17	(0%)
Acute Myeloid Leukemia	0/15	(0%)
Bladder	0/5	(0%)
Glioma	0/9	(0%)
Hepatocellular	0/7	(0%)
Kidney	0/6	(0%)
Melanoma	0/17	(0%)
Non-Small Cell Lung	0/25	(0%)
Thyroid	0/1	(0%)

Supplementary Table 5. List of primers.

Primers for Bisulfite Genomic Sequencing		
DERL3	Fr	TTGGTTGTYGGGTTTAGG
	Rv	ACCAAAAACCAATCAAAAACT
DERL1	Fr	GTATTAAAATTTTGAGAATTAGGTGAA
	Rv	AACCAACRAAACAACCTTCTA
DERL2	Fr	ATGTGYGTTTTTTAGGTTTTTA
	Rv	AATTTCCTAAAAACCCAATCAT
Primers for Methylation-Specific PCR		
DERL3	Fr Unmethylated	GAGTTTTTGTAGGTGTTGGT
	Rv Unmethylated	CCATAACCATAACAACCCT
	Fr Methylated	GAGTTTTTGTAGGTGTCGGC
	Rv Methylated	CCGTAACCATAACGACCCT
Primers for qRT-PCR		
DERL3	Fr	CCTCGTGGACCTGCTGG
	Rv	GGTCTGCAGGAGCCTCTTG
B2M	Fr	CCTGAATTGCTATGTGTCTGG
	Rv	CTCCATGATGCTGCTTACATGTCTCG
SLC2A1	Fr	CATCAACGCTGTCTTCTATTACTC
	Rv	ATGCTCAGATAGGACATCCA
Primers for DERL3 cloning		
DERL3-XhoI	Fr	CTCGAGCGCCACCATGGCGTGGCAGGGACTAGCGGCCGAGTTCCTGCAGGTGCCGGCGGTGACGC
DERL3-FLAG-GSG-BamHI	Rv	GGATCCTCACTTATCGTCGTCATCCTTGTAATCGCCGGAGCCCTGCTGCGGGGGTGGCAGATGGGGTCC TGGCTGTTCCTCAGGGAGGGGCAGGTAATTGGG

Fr: Forward primer

Rv: Reverse primer
