# Supplementary material for: Corrigendum: A DERL3-associated defect in the degradation of SLC2A1 mediates the Warburg effect
Source: Nat Commun. 2016 Nov 2;7:13467. doi: 10.1038/ncomms13467 (PMC5097131; doi:10.1038/ncomms13467)

Supplementary Figure 1. Unprocessed gel image of DERL3 methylation-specific PCR related to Figure 1b.

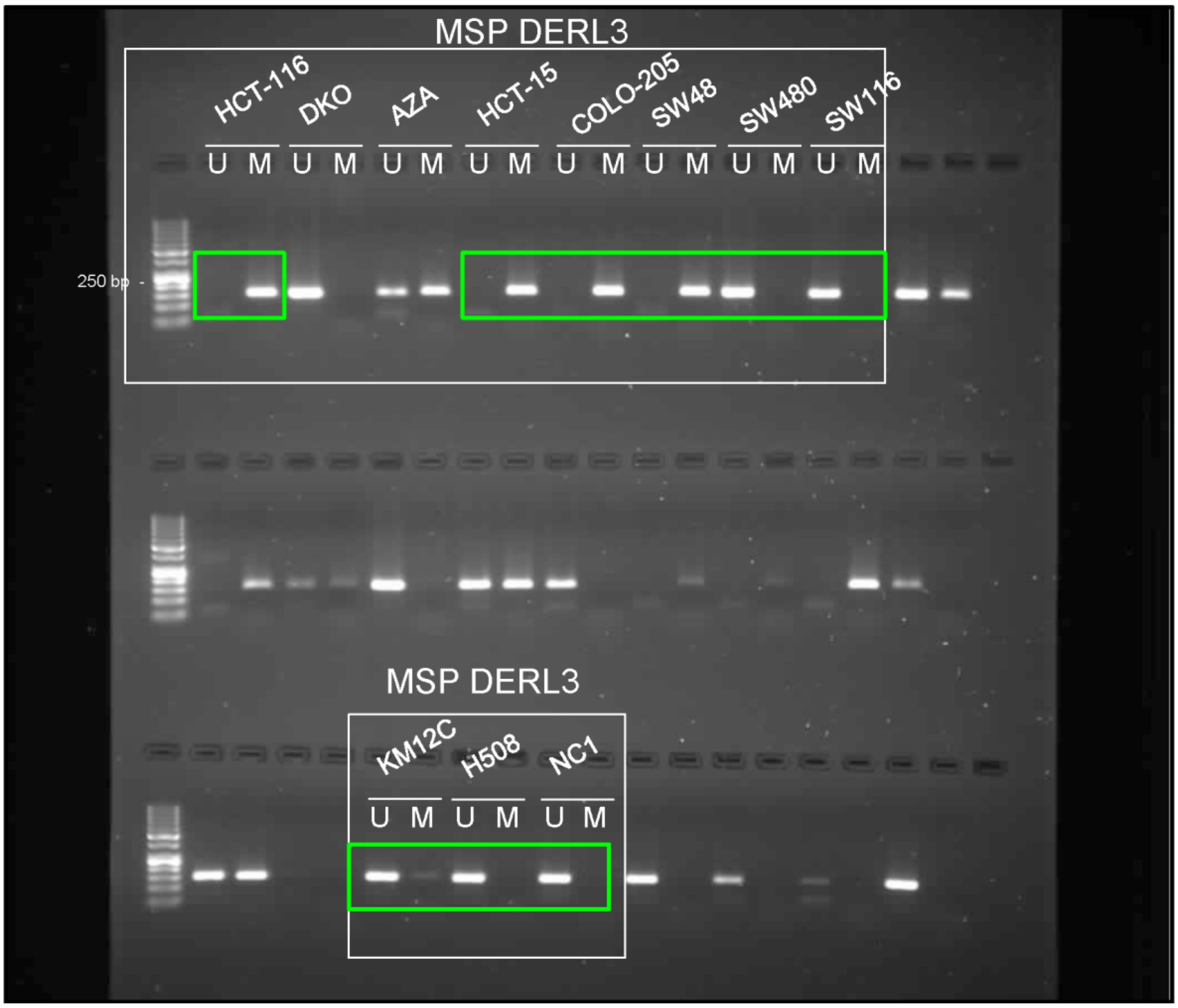

Supplementary Figure 2. Unprocessed gel image of DERL3 methylation-specific PCR related to Figure 1b.

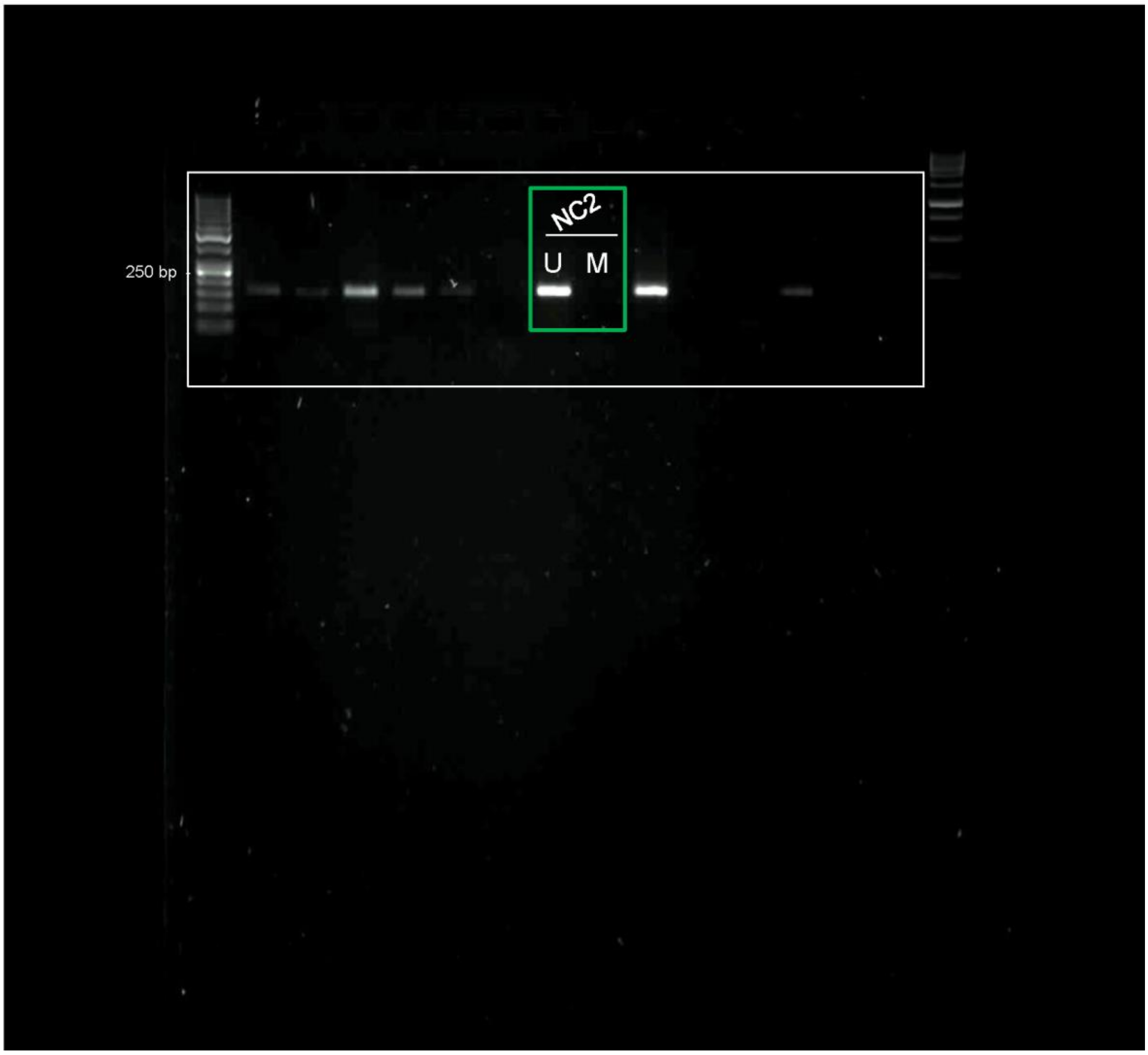

Supplementary Figure 3. Unprocessed gel image of DERL3 methylation-specific PCR related to Figure 1b.

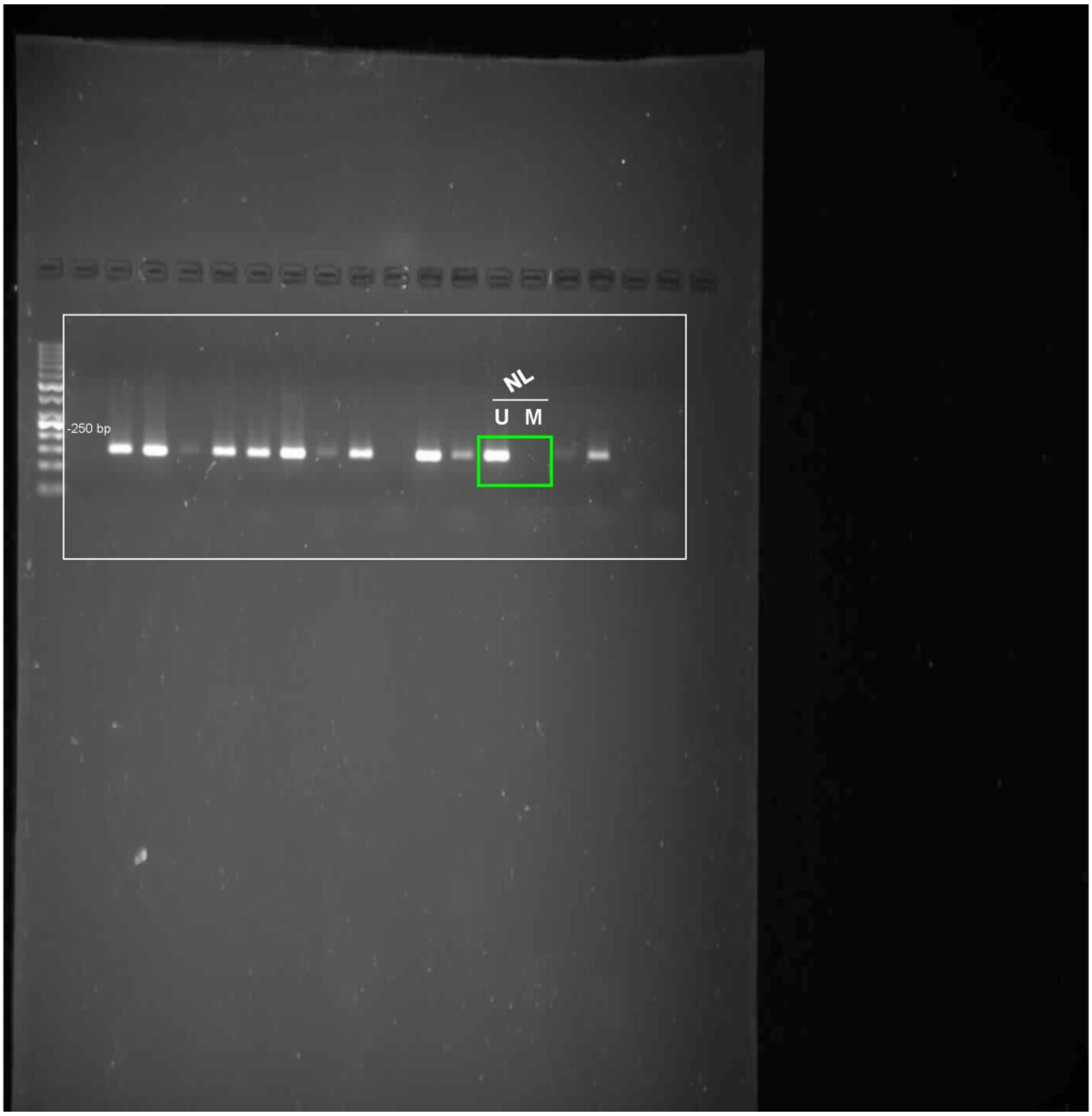

Supplementary Figure 4. Unprocessed gel image of DERL3 methylation-specific PCR related to Figure 1b.

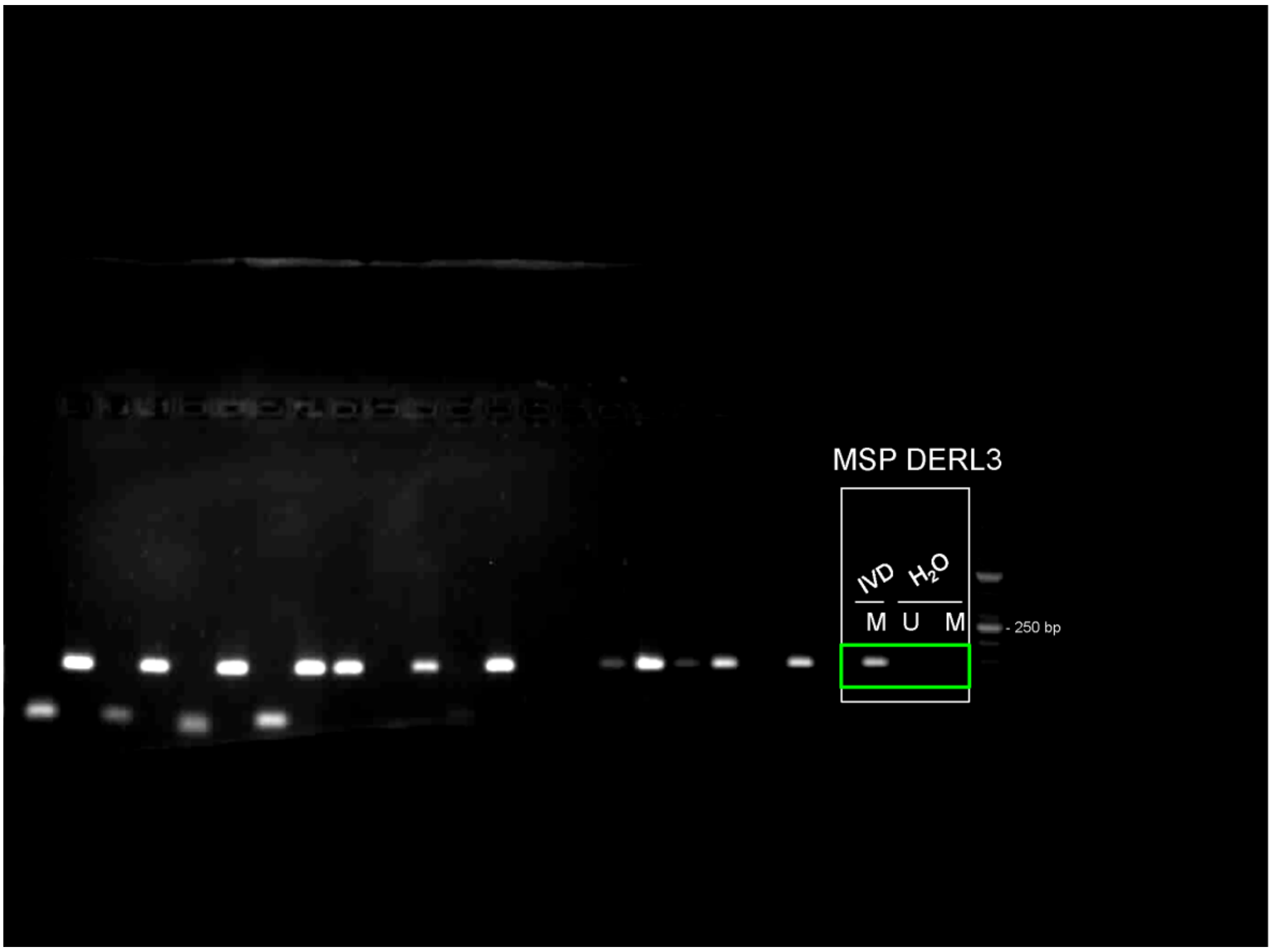

Supplementary Figure 5. Unprocessed gel image of DERL3 RT-PCR.

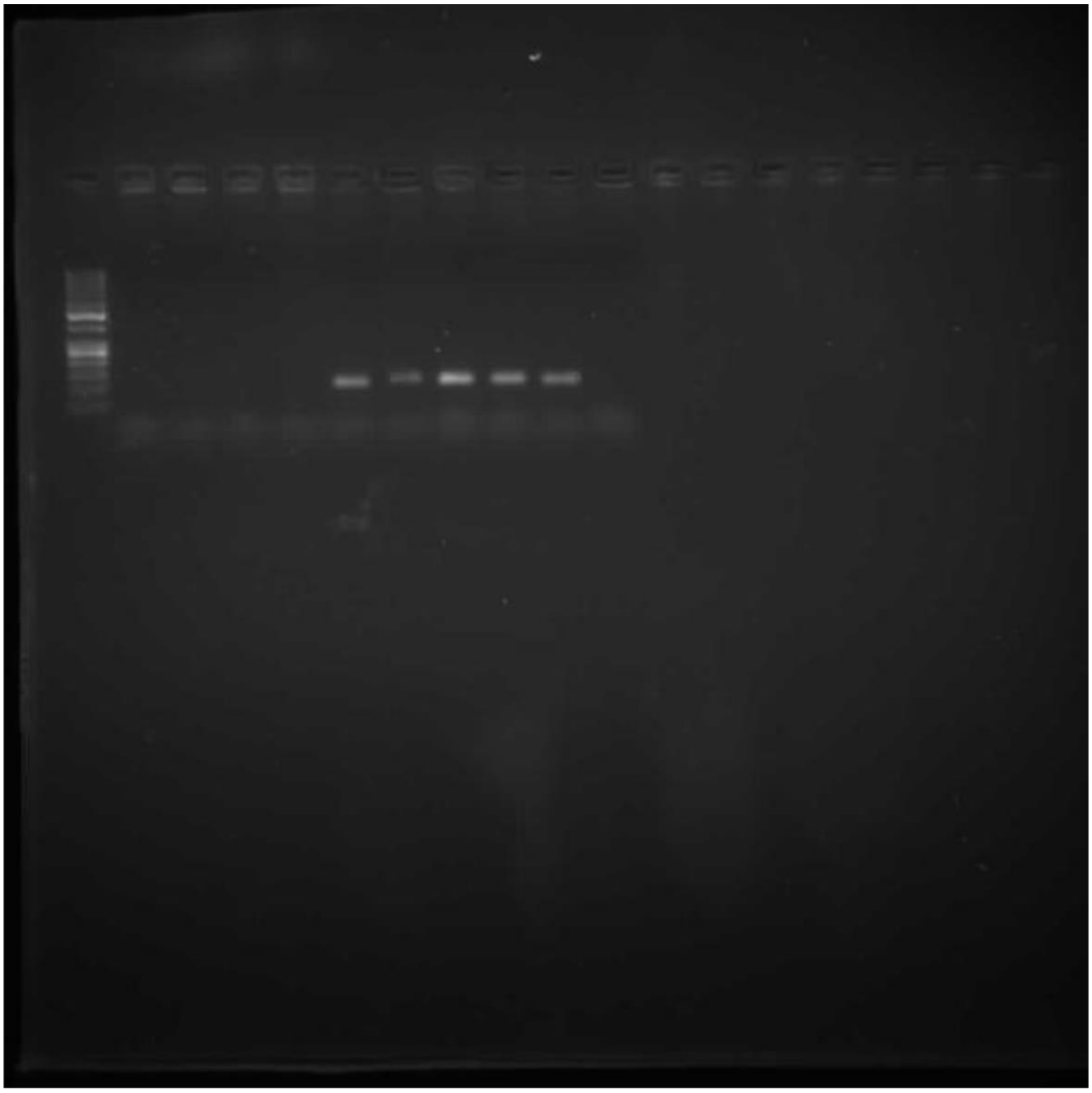

Supplementary Figure 6. Unprocessed gel image of B2M RT-PCR.

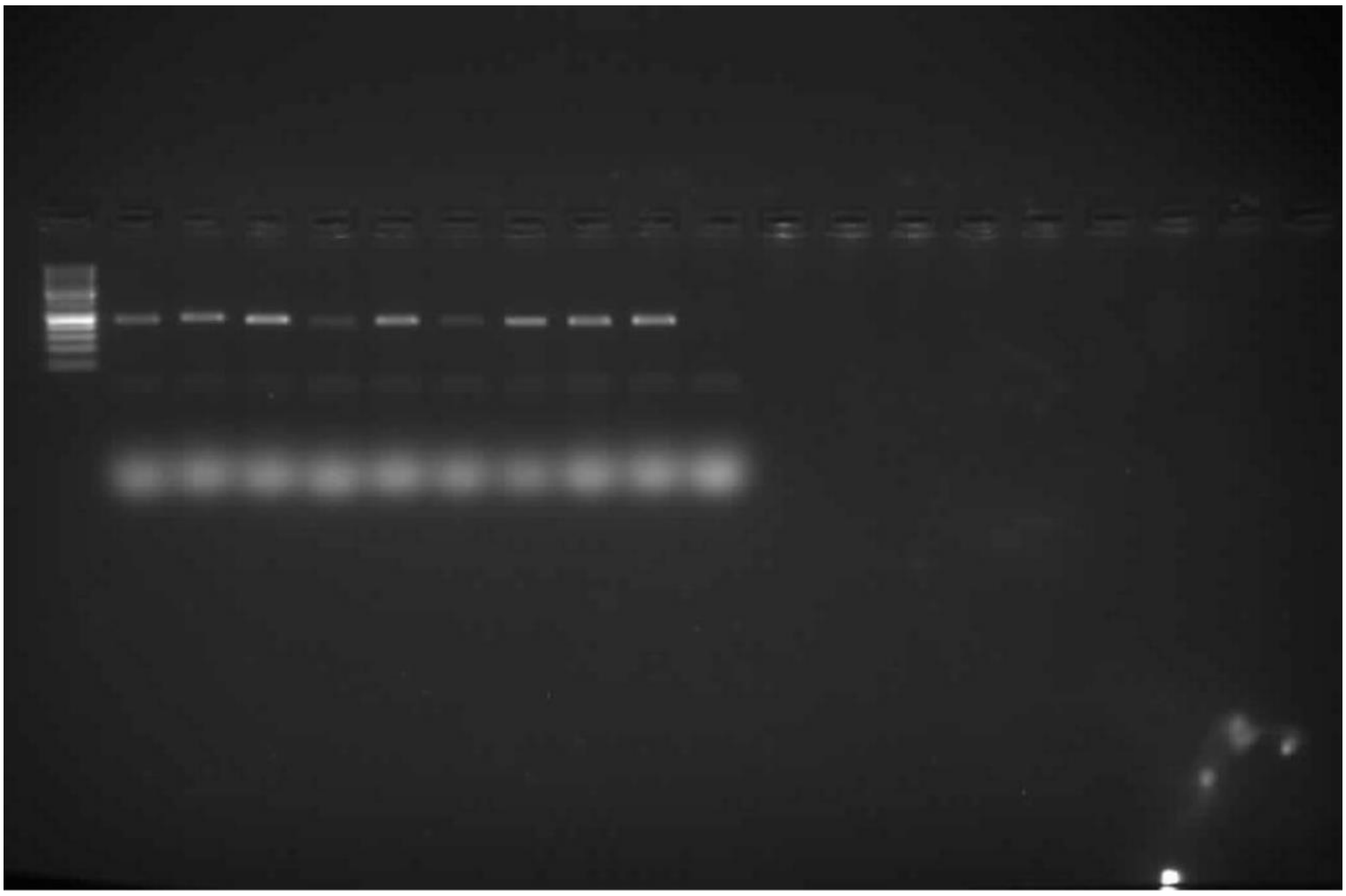

Supplement: Supplementary file 1 [file ncomms13467-s1.pdf]
